# Psychometric evaluation of the depression, anxiety, and stress scale-21 (DASS-21) among Chinese primary and middle school teachers

**DOI:** 10.1186/s40359-023-01242-y

**Published:** 2023-07-14

**Authors:** Cui-Hong Cao, Xiao-Ling Liao, Xing-Yong Jiang, Xu-Dong Li, I-Hua Chen, Chung-Ying Lin

**Affiliations:** 1 Faculty of Education, Qufu Normal University, Qufu, 273165 China; 2 School of Foreign languages, Shandong Women’s University, Jinan, 250300 China; 3 Faculty of Education, Jiangxi Science and Technology Normal University, Nanchang, 330031 China; 4 Yangan Primary School of Qionglai City, Qionglai, 611535 China; 5 Gaogeng Nine-year School, Qionglai, 611533 China; 6 Chinese Academy of Education Big Data, Qufu Normal University, Qufu, 273165 China; 7 Institute of Allied Health Sciences, College of Medicine, National Cheng Kung University, Tainan, 701401 Taiwan, ROC; 8 Biostatistics Consulting Center, College of Medicine, National Cheng Kung University Hospital, National Cheng Kung University, Tainan, 701401 Taiwan, ROC

**Keywords:** Psychometric properties, The DASS-21, School teachers, Rasch model, Network analysis

## Abstract

**Background:**

Teachers in high-stress roles face increasing psychological distress such as anxiety and depression, underscoring the need for validated assessment instruments. Given the current absence of a comprehensive, designated, and time-efficient scale capable of evaluating depression, anxiety, and stress among the teacher population, the Depression, Anxiety, and Stress Scale-21 (DASS-21) presents itself as a promising alternative. Despite the widespread application of the DASS-21 for assessing psychological distress across various populations, its validity among teachers, along with questions about its factor structure and its potential property of time equivalence, remain unverified. This study endeavors to address these considerations by investigating the psychometric properties of the DASS-21 specifically within the population of Chinese primary and middle school teachers.

**Methods:**

Cross-sectional (n = 9,030) and longitudinal surveys (n = 1,642) were conducted using a non-probability sampling method. In addition to the DASS-21, the Chinese version of Chinese Teachers’ Job Burnout Questionnaire (CTJBO) was utilized to evaluate the criterion validity of this scale. Three different approaches, namely confirmatory factor analysis, Rasch analysis, and network analysis, were employed to evaluate internal reliability, construct validity, as well as time invariance of the DASS-21.

**Results:**

The DASS-21 demonstrated a high degree of internal consistency (Cronbach’s α > 0.85) as well as excellent convergent validity, despite poor discriminant validity as determined by average variance extracted. Confirmatory factor analysis and network analysis further supported convergent validity. The three-factor structure outperformed one- and two-factor alternatives, establishing time invariance. Rasch analysis at the item level identified six inappropriate items within the anxiety and stress subscales, which were subsequently removed. Network analysis presented a better revised network. Regression analysis with emotional exhaustion as the criterion provided logical and accurate results.

**Conclusion:**

The DASS-21 was found to be a reliable and valid tool for measuring the mental health of teachers over time. To assess the instrument’s psychometric properties, a combination of confirmatory factor analysis, Rasch analysis, and network analysis was utilized, which proved effective and is recommended for evaluating contentious instruments. Based on the results of the study, researchers and healthcare professionals are recommended to use the DASS-21 for assessing teachers’ psychological distress. However, certain items identified in the study may need to be removed to enhance the instrument’s appropriateness for this specific population.

**Supplementary Information:**

The online version contains supplementary material available at 10.1186/s40359-023-01242-y.

## Background

Teaching has been reported as a comparatively high-stress occupation when compared to other professions in numerous studies [1–4]. Teacher stress, as a particular type of occupational stress, refers to the psychological discomfort and disorder that teachers experience both mentally and physically in the school environment [4]. It can be attributed to multiple factors such as difficulty dealing with interpersonal relationships, and administrative pressure [5, 6]. Besides, the increasing use of information technology and globalization has made teaching more demanding in recent years. The mandatory requirements for technology integration without sufficient technical resources and training has become another potential source of stress [7, 8]. In addition, excessive workload, disruptive student behavior, and unrealistic expectations were identified as the three main sources of stress commonly experienced by teachers in Collie and Mansfield’s study [9]. Furthermore, García-Carmona et al.’s systematic review and meta-analysis underscores several risk factors contributing to teacher stress. These encompass issues such as unsatisfactory income, time pressure, overcrowded classrooms, inadequate facilities, and a dearth of training or promotional opportunities [10]. Teachers are consequently burdened by these myriad sources of stress.

Prior research indicates that stress not only undermines teachers’ mental and physical health but also engenders negative emotional disorders such as anxiety and depression, thereby impeding their work productivity and capacity to handle daily tasks [11]. Skaalvik and Skaalvik [12] demonstrated that teacher-experienced stress could instigate anxiety, foster feelings of incompetence, and necessitate self-protection. Moreover, stress has been implicated in the development of depression [13]. In a meta-analysis, Montgomery and Rupp scrutinized the causes and effects of stress among teachers, concluding that stress was correlated with emotional responses in this population, such that experiences of stress evoked distress, anxiety, and depression [14].

For teachers, such psychological distress can engender deleterious consequences. They may lead to lower job satisfaction, life satisfaction and increased their attrition rates [2, 6]. Those who choose to stay in the profession may experience decreased motivation and teaching efficacy [15]. As teachers are critical roles in the educational system, taking care of their mental health is important because their poor mental health can significantly influence educational outcomes [16]. Mental disorders among teachers pose a major challenge as they are associated with various variables that can impact a student’s academic success. Prior research has shown that teachers’ mental health is strongly linked to student behavior and can bring about poor student academic achievement and damaged teacher-student relationships in the long run [17]. As a result, to measure teachers’ mental health, a reliable and valid scale is urgently needed.

The majority of quantitative studies examining the mental health of Chinese teachers employ the Symptom Checklist 90-Revised (SCL-90-R), a self-report measure, as the instrument of choice [18]. The SCL-90-R encompasses 90 items, subdivided into nine dimensions, including depression and anxiety. Yang et al.’s meta-analysis concluded that while the SCL-90-R is commonly used among teachers, the findings of empirical studies using it are inconsistent: some indicate a good state of teachers’ health, while others suggest poorer health conditions compared to the general population [18]. Moreover, questions have been raised regarding the clinical validity of many of the SCL-90-R’s symptoms [19]. Furthermore, the 90-item scale is time-consuming and lacks efficiency. In light of these shortcomings, this study introduces a different scale for evaluation within the teacher population.

The Depression, Anxiety, and Stress Scale which is shortened as DASS is a widely used mental health assessment tool. The original 42-item full version is divided into three subscales: Depression, Anxiety, and Stress [20]. The instrument, developed by Lovibond & Lovibond in 1990s, was designed to recognize and distinguish common mental disorders using standardized measurement system [21]. It aimed to supply clinical diagnosis with psychometric indicators and serve as a rapid, efficient measurement instrument for related studies [20]. Later on, the full version of DASS-42 was shortened to be a 21-item version (i.e., the DASS-21), a version retains all dimensions of the original scale while enhancing the identification and evaluation of emotional disorders based on corresponding symptoms.

Due to its simplicity, novelty, uniqueness, and convenient operation, the DASS-21 has gained popularity. Different versions have been translated and validated for research and application worldwide, including Arabic [22], Bangla [23], Chinese [21], Hindi [24], Malaysian [25], Persian [26], etc. Moreover, it has also been utilized to assess the psychological distress symptomatology of diverse populations, including university students [27], athletes and non-athletes [28], adolescents [29], parents of young children [30], as well as teaching staff [31].

Previous studies have used the DASS-21 among teachers, including primary and secondary schoolteachers in the USA and mainland China [32, 33]. However, to our knowledge, the current literature does not have psychometric testing studies reporting the DASS-21’s reliability and validity for assessing psychological distress among teachers. Moreover, there is no evidence to indicate its factor structure is time invariant. In addition, different factor structures with regard to this scale have been reported: Some studies have supported a one-factor structure [34], or bifactor structure [28, 35], while others are in favor of the traditional oblique three-factor structure [36, 37].

Moreover, in terms of psychometric approaches, previous studies mainly adopted confirmatory factor analysis (CFA) (e.g., using CFA to assess the factorial validity in [26, 27]). Prior psychometric evidence of the DASS-21 using Rasch measurement model [38, 39] or network analysis [40] was established on populations other than teachers. Therefore, it is unclear if Rasch analysis and network analysis results of the DASS-21 can be replicated on teacher population. Given that psychometric evidence of an instrument needs to be cumulated using different statistical approaches, simultaneously using CFA, Rasch model, and network analysis could provide strong evidence for the psychometric properties of the DASS-21. The Rasch measurement model, also known as the Rasch analysis, was originally proposed by the Danish mathematician Georg Rasch [41]. It is an item response theory model that characterizes each item based on a single parameter, the item difficulty [42]. The analysis is based on the idea that a respondent’s ability, as well as the item’s difficulty, determines the chances of correctly answering an item [43]. Additionally, the Rasch analysis can be used to refine item checks through item addition or removal [44]. By analyzing the data rigorously and extensively, the Rasch analysis can provide additional psychometric information not available through traditional approaches [45]. To the best of our knowledge, the Rasch analysis has been applied to the DASS in two studies [38, 39]. In Shea et al.’s study, Rasch analysis was conducted to the DASS-21 and it was suggested removing one item from each subscale in order to achieve an adequate model fit for each subscale [38]. In contrast, Medvedev et al. found that non-fitting item 5 should be removed after conducting Rasch analysis to achieve the best model fit [39]. However, neither study included teachers as participants nor tested the time invariance of the DASS-21.

The network analysis is to present information about a particular system in the form of a network, which is composed of nodes and edges [46]. As a method of visualizing the complex relationships between symptoms on the basis of the dynamic systems modeling [47], the network analysis can be used to represent, analyze, and study systems in their full complexity [48]. Van den Bergh et al. conducted a network analysis of the DASS-21 symptoms in a large number of international respondents, revealing that certain symptoms can connect three subscale clusters, leading to a better understanding of comorbidity and reciprocal influences [49].

Given that CFA, Rasch analysis, and network analysis each offer distinct insights in the field of psychometrics due to their unique focal points, simultaneously reporting findings from the three different statistical analyses could provide a comprehensive picture for researchers to better understand the properties of a psychometric instrument. Other benefits of using three methods simultaneously include (i) having the comparable findings from the three methods because the same sample was used for the psychometric testing and (ii) some psychometric properties of the instrument (e.g., unidimensionality for each DASS-21 subscale) could be verified using different methods. However, most studies have typically employed a combination of two out of the three methodologies to thoroughly evaluate the reliability and validity of scales, as exemplified by research such as [25, 40]. In the context of the DASS-21—a scale that has been subject to considerable debate regarding its factor structure [34–37]—the provision of three types of psychometric testing in one study (like the present one) is informative. Specifically, CFA can serve to validate the theoretical structure, addressing the controversy surrounding the scale’s factorial validity [26, 27]. Rasch Analysis provides important insights into the functionality of individual items, evaluating item validity in line with previous studies of the DASS-21 [38, 39]. This approach extends these insights to include a teacher population sample, and it also helps to identify any potentially misfitting items. Following this, network analysis uncovers complex relationships among items that extend beyond the underlying constructs [49]. After item diagnosis via Rasch analysis, network analysis, through its visual representation of nodes and edges, offers an intuitive understanding, allowing for a visual comparison between the original and any potentially revised structures of the DASS-21. This combined application of the three methodologies ensures a robust and versatile psychometric examination, enhancing our understanding of the DASS-21 and its potential applications.

To summarize, the literature review has identified two research gaps. Firstly, the SCL-90-R, which is currently used to measure teachers’ mental health, exhibits several deficiencies. In addition, the DASS-21, although validated among different populations, has not had its psychometric properties specifically tested with respect to teachers as a demographic. Therefore, it is of significant importance to evaluate the DASS-21 among teachers. Another identified gap is that previous studies evaluating the DASS-21’s psychometric properties among different populations or in different languages have not combined three approaches - CFA, the Rasch analysis, and network analysis - in a single study. To address these gaps, the main purpose of the present study was to assess the DASS-21’s psychometric properties (i.e., internal reliability, construct validity, as well as time invariance) among primary and middle school teachers using the aforementioned approaches successively. Additionally, we aimed to test the criterion validity of this scale by examining its association with emotional exhaustion. According to the Stressor–Strain–Outcome (SSO) model [50], emotional exhaustion indicates strain, while psychological distress is an outcome. These two are closely related, and many empirical studies on teachers have confirmed their significant correlation [51, 52].

## Methods and procedures

### Participants

The present study comprised a cross-sectional survey and a longitudinal survey, both of which adhered to the same inclusion criteria: (1) being a primary or middle school teacher; (2) being able to read and write in Chinese; (3) providing electronic informed consent. The shared exclusion criterion was: non-participation in school work due to illness or other reasons. A non-probability sampling strategy was adopted for both surveys.

The cross-sectional survey was conducted online among primary and middle school teachers using an online questionnaire collection platform (i.e., Sojump) from May to June in 2020. Three provinces—Sichuan, Jiangxi, and Shandong, representing three parts of mainland China - the western, central, and eastern parts- respectively, were selected. These three provinces together account for half of the nation’s population. We contacted the principals who are in charge of primary and middle schools, informed them of the study’s purpose and duration, and assured them that participation was voluntary and anonymous, and all data collected would be used for research purposes only. Only after obtaining electronical informed consent did we proceed with the study. In the end, we obtained a total of 9,030 valid questionnaires.

The longitudinal survey was also conducted online in a city located in a central province of China. With the strong support and assistance from local education authorities, we carried out two waves of online surveys among research subjects. The first wave was conducted in November 2021 (Wave 1), two weeks after emergency remote teaching implementation during the COVID-19 pandemic, and the follow-up survey was carried out two months later in January 2022 (Wave 2) when campuses had reopened, and in-person classes had been in place for two weeks. The interval was set in this way to better detect the mental health state of teachers during and after the pandemic emergency remote teaching. The education administration staff transmitted the online questionnaire hyperlink to primary and middle school teachers within their jurisdiction. During the first wave, participants who were interested in taking part in the follow-up survey two months later were invited to leave their email addresses. On the first page of the questionnaire, we informed participants of our purpose, the researcher’s affiliation, and guaranteed that the collected data would be stored and curated appropriately to ensure privacy. After obtaining electronical informed consent, we continued with the online survey. Ultimately, 1,642 teachers completed both waves of the online survey.

The present study got approval from the Institutional Review Board (IRB) of the Jiangxi Psychological Consultant Association (IRB ref: JXSXL-2020-J013). Please refer to the Results section below for information on the participants’ characteristics.

### Measures

#### The depression, anxiety, and stress scale-21 (DASS-21)

We adopted the Chinese version of the Depression Anxiety and Stress Scale-21 (DASS-21) to measure psychological distress among primary and middle school teachers in the present study. The DASS-21 consists of three subscales, namely depression, anxiety, and stress, each consisting of 7 items [20]. Example items for each subscale are: “I was unable to become enthusiastic about anything” (depression); “I felt scared without any good reason” (anxiety); and “I tended to over-react to situation” (stress). Each item is rated on a four-point Likert scale, indicating how the statement applied over the past week to the respondents [53]. The higher total score means that the psychological distress is more severe. The DASS-21 has been extensively applied in Chinese samples [21, 54], and previous studies have shown good internal consistency in teaching staff [32, 33]. In this study, the DASS-21 of the Chinese version was demonstrated with high internal consistency in both the cross-sectional and longitudinal surveys (Cronbach’s α for all the three subscales ranging from 0.86 to 0.92). The cut-off score indicating the presence of clinical depression, anxiety, and stress is 5 or more, 4 or more, and 8 or more respectively [20].

#### Chinese teachers’ job burnout questionnaire (CTJBO)

With the purpose of evaluating the criterion validity of the DASS-21, we chose to utilize the subscale called Emotional exhaustion in the Chinese version of CTJBO to measure emotional exhaustion among primary and middle school teachers during Wave 2 of the longitudinal study. This scale was revised by Wu et al. [55] from the Maslach Burnout Inventory (MBI) [56] to meet Chinese culture. The revised 22-item questionnaire consists of 3 dimensions. Besides Emotional exhaustion (8 items) that we adopted in the present study, the other two are Depersonalization (8 items) and Personal accomplishment (6 items) [55]. All items are scored on a seven-point Likert scale to test whether the respondent feels the same way about one’s job from 1 (never) to 7 (every day) [56]. A sample item is “I feel emotionally drained from my work”. Wu et al. found that the CTJBO demonstrated good construct validity and reliability, and that job burnout was positively correlated with depression [55]. Being a significant factor of burnout, emotional exhaustion was selected as the criterion to test the validity of the DASS-21. The internal reliability of the adopted Emotional exhaustion subscale was good (Cronbach’s α = 0.95).

### Analysis strategy

#### Descriptive statistics, test-retest reliability and CFA

Descriptive statistics were adopted by the present study to present the participants’ characteristics and the level emotional exhaustion reported. Additionally, the prevalence of clinical depression, anxiety, and stress was determined using cutoff points provided by Lovibond and Lovibond [20]. Meanwhile, intraclass correlation coefficients (ICC) were used to assess the test-retest reliability. Subsequently, the classical test approach – CFA using diagonally weighted least squares (DWLS) estimation, given the ordinal response options – was conducted to test the factorial validity. This included one-factor, two-factor (depression and a combined factor of anxiety with stress) [57], and three-factor models [20]. The criteria adopted to evaluate model fit include: comparative fit index (CFI) and the non-normed fit index (NNFI) greater than 0.90; root mean square error of approximation (RMSEA) less than 0.06 and standardized root mean square residual (SRMR) less than 0.08 [58]. In addition, Akaike information criterion (AIC) and ΔAIC (AIC higher - AIC lower) were used to determine the best-fitting model, with a smaller AIC indicating a better fit [59]. As a further step, convergent and discriminant validity were assessed using composite reliability (CR) and average variance extracted (AVE). For each construct, evidence of convergent validity was considered present if CR was greater than 0.70 and AVE was greater than 0.50 [60]. To ensure discriminant validity, the square root of the AVE of the construct must exceed the correlation between it and other latent variables [60]. Lastly, time invariance across different assessment periods was evaluated using various models. We used the method introduced by Wu and Estabrook [61], which asserts that the traditional approach for testing measurement invariance, which involves setting a baseline model and imposing increasing restrictions on loadings and thresholds, is not the optimal for ordered categorical variables. Instead, they propose an alternative process. After creating the configural model, thresholds should be constrained first, followed by restrictions on factor loadings. Following this approach, our test procedures included: (1) Creating a configural invariance model, (2) Forming a threshold invariant model, (3) Constructing a model with both threshold and factor loading invariance, (4) Building a model with threshold, factor loading, and intercept invariance, and (5) Establishing a model with invariance in threshold, factor loading, intercept, and residual variance. We compared the different models using changes in CFI (ΔCFI), RMSEA (ΔRMSEA), and SRMR (ΔSRMR). Invariance was supported by the following criteria: ΔCFI greater than − 0.01, ΔRMSEA less than 0.015, ΔSRMR less than 0.03 (for factor loading constrained) or less than 0.01 (for item threshold constrained) [62].

#### Rasch analysis

Rasch analysis was conducted using the following strategies. Firstly, to determine whether to use the Andrich Rating Scale Model (RSM) or the Partial Credit Rasch Model (PCM), a likelihood ratio test was conducted. It was found that the PCM should be used as it was statistically significant. Next, the threshold ordering (category structure) of each item was examined to check whether the category calibration increases in an orderly manner. We selected the visual method using Person-Item map to scrutinize the functionality of the response categories. Additionally, the requirement of uni-dimensionality for Rasch analysis and the person fit to the model was assessed using the following criterion: in terms of the uni-dimensionality, eigenvalue ratios [i.e., the ratio of the first to second eigenvalues by the principal component analysis (PCA)] higher than 4.00 [63] and the eigenvalue of the first component in the PCA of the residuals less than 2.00 [64]; for a good model fit, the standard deviation of the mean persons’ residuals should be less than 1.50 [38, 65]. Moreover, person reliability was also evaluated with the acceptable criterion being higher than 0.70 [41].

For the diagnostic items, in addition to point-biserial correlation of each item, information-weighted fit statistic (infit) mean square (MnSq), and outlier-sensitive fit statistic (outfit) MnSq were also used. For the point biserial correlation of each item, a positive direction with the value between 0.30 and 0.70 is expected and this reflects the item potentially contribute to the latent variable [66, 67]. Acceptable good fit values for infit/outfit ranged from 0.7 to 1.3 [68]. If the infit/outfit value was below 0.7, it meant that the item provided no additional information beyond what was already given by the other items on the scale, which could happen due to the existence of similar items or a high degree of correlation among items, or when one item was contingent on another. Conversely, if the infit/outfit value was above 1.3, it indicated that this item did not capture same construct of the other items, and was either poorly constructed, poorly understood, or ambiguously defined. Consequently, items with subpar fit statistics should be considered for elimination from the scale [68].

#### Network analysis

To examine the impact of removing inappropriate items by Rasch analysis (if necessary) on the findings of network analysis, the next step involved visualizing the results. The network was constructed using the Extended Bayesian Information Criterion Graphical Least Absolute Shrinkage and Selection Operator (EBICglasso). A tuning parameter of 0.5 was set to generate a more parsimonious and straightforward network with fewer connections, greater specificity, and higher sensitivity. Nodes (i.e., items) were connected using edges, with thickness indicating strength, and blue and red colors denoting correlations are positive and negative, respectively. To evaluate the centrality of nodes, we computed measures of betweenness (connectivity), closeness (distance centrality), strength (degree centrality), and expected influence (a node’s cumulative impact on a network, representing the activation, persistence, and remission of the node in the network), as recommended by previous studies [69, 70]. Before presenting the centrality results, we checked the stability and accuracy of the network. Centrality stability was determined using case-dropping subset bootstrapping, which analyzes whether centrality indices remain constant across different subsets [71]. The correlation stability coefficient (CS-coefficient) was used to determine the stability of centrality, with a value above 0.5 preferred and a value below 0.25 considered unstable and uninterpretable [71]. Additionally, non-parametric bootstrapped samples were used to calculate 95% confidence intervals (CIs) to determine accuracy, with narrower CIs indicating more accurate edge estimates [71].

#### Criterion validity

A regression analysis was conducted to examine DASS-21’s criterion validity with emotional exhaustion. The model included teachers’ emotional exhaustion as the dependent variable and factor scores of the three subscales as the explanatory variables, along with control variables such as sex and age. The analysis also involved a comparison between the original DASS-21 and a modified version obtained by removing any inappropriate items identified by Rasch analysis. These steps helped ensure the accuracy and reliability of our findings.

## Results

Table 1 displays participants’ characteristics in both surveys. The cross-sectional survey was conducted between May and June 2020, and the second longitudinal survey was conducted in November 2021 (Wave 1) and January 2022 (Wave 2), with a two-month interval. For the cross-sectional survey, 9030 participants were recruited, of which 5838 (64.65%) were from primary school, and female occupied the vast majority (6563, 72.7%). They taught different subjects, with Chinese literature being the most followed by English. Based on the DASS-21, the number of teachers with probable depression was 1843 (20.41%), probable anxiety was 2388 (26.44%), and probable stress was 917 (10.15%). For the longitudinal survey, 1642 participants were recruited, with the majority from primary school (1159, 70.6%), and a high proportion of female teachers (1305, 79.5%). As for teaching subject, Chinese literature was the most followed by Mathematics. The DASS-21 score for Wave 1 showed that the number of teachers with probable depression was 368 (22.41%), probable anxiety was 589 (35.87%), and probable stress was 265 (16.14%). In Wave 2, the number of teachers with probable depression was 386 (23.51%), while the other two decreased slightly to 570 (34.71%) for anxiety and 258 (15.71%) for stress. The score of teacher emotional exhaustion was 3.46. Moreover, ICC (2,1) of depression, anxiety, and stress subscales was 0.73, 0.71, and 0.71, demonstrating acceptable test-retest reliability.

**Table 1:** The characteristics of the participants

Source	Cross-sectional survey	Longitudinal survey	
Data collection period	2020.5-6	2021.11 (Wave 1)	2022.01 (Wave 2)
Valid number	9030	1642	
School type (Primary school); n (%)	5838 (64.65%)	1159 (70.6%)	
Sex (Female); n (%)	6563 (72.7%)	1305 (79.5%)	
Age; Mean (SD)	33.94 (8.81)	34.22 (8.72)	
Teaching subject; n (%)			
Chinese literature	3624 (40.13%)	638 (38.86%)	
English as foreign language	1601 (17.73%)	198 (12.06%)	
Mathematics	3222 (35.68%)	533 (32.46%)	
Others (science, social science, music, art, physics, politics)	583 (6.46%)	273 (16.62%)	
The DASS-21; n (%)			
Probable Depression	1843 (20.41%)	368 (22.41%)	386 (23.51%)
Probable Anxiety	2388 (26.44%)	589 (35.87%)	570 (34.71%)
Probable stress	917 (10.15%)	265 (16.14%)	258 (15.71%)
Teacher Emotional Exhaustion (range:1–7); Mean (SD)	Not applicable	Not applicable	3.46 (1.51)

Note. Probable is determined according to the cutoff point by Lovibond and Lovibond

Table 2 presents the comparison of the model fit between different factor structures. The results showed that all three factor structure types were acceptable for participants in both cross-sectional and longitudinal surveys, including two waves of data (CFI = 0.992 to 0.996; NNFI = 0.991 to 0.996; RMSEA = 0.049 to 0.063; and SRMR = 0.029 to 0.045). Although all three factor structures had acceptable model fits, the three-factor model outperformed the other two factor structures across all studies. Specifically, the ΔAICs for the three-factor model compared to the other two factor models were all greater than 10, indicating that the three-factor model was strongly supported by the empirical data (see Table 2).

**Table 2:** Results of CFA Model Fit

	*χ*^*2*^ (*df*)	CFI	NNFI	RMSEA (90% Confidence Interval)	SRMR	AIC
**One-factor structure**						
Cross-sectional survey	4336.87 (189)	0.996	0.996	0.049 (0.048–0.051)	0.029	4420.87
Longitudinal survey Wave 1	1422.98 (189)	0.992	0.991	0.063 (0.060–0.066)	0.045	1506.98
Longitudinal survey Wave 2	1290.92 (189)	0.994	0.993	0.060 (0.057–0.063)	0.039	1374.92
**Two-factor structure**						
Cross-sectional survey	4339.81 (188)	0.996	0.996	0.049 (0.048–0.051)	0.029	4425.81
Longitudinal survey Wave 1	1430.97 (188)	0.992	0.991	0.063 (0.060–0.067)	0.044	1516.97
Longitudinal survey Wave 2	1270.55 (188)	0.994	0.993	0.059 (0.056–0.062)	0.039	1356.55
**Three-factor structure**						
Cross-sectional survey	4288.06 (186)	0.996	0.996	0.049 (0.048–0.051)	0.029	4378.06
Longitudinal survey Wave 1	1373.08 (186)	0.992	0.991	0.062 (0.059–0.066)	0.044	1463.08
Longitudinal survey Wave 2	1226.00 (186)	0.994	0.994	0.058 (0.055–0.062)	0.038	1315.99

CFI = comparative fit index; NNFI = non-normed fit index; RMSEA = root mean square error of approximation; SRMR = standardized root mean square residual

Following the confirmation that the three-factor structure had the best model fit, the factor loading in this model was used to calculate CR and AVE for evaluations of convergent validity (see Table 3). The results showed that CR was higher than 0.90 and AVE was greater than 0.60, indicating excellent convergent validity. However, there was a lack of discriminant validity across all subscales of the DASS-21 due to the overly high correlations between each pair of factors (see Table S1). In order to test the time-invariant property of the DASS-21, the configural model and the respective nested models were compared after confirming that the three-factor structure was acceptable for both waves in the longitudinal study. Based on the results presented in Table 4, we can conclude that the DASS-21 exhibits full time-invariant properties, specifically the equivalence of thresholds, factor loadings, intercept, and residual variances, across two-month intervals. This conclusion is supported by the fact that the ΔCFI, ΔRMSEA, and ΔSRMR all meet the specified criterion.

**Table 3:** Item properties in the DASS-21 across two surveys of the participants

Source	Cross-sectional survey (N = 9030)		Longitudinal survey (N = 1642)						
The Depression, Anxiety and Stress Scale − 21			Wave 1				Wave 2		
Subscale of Depression	Mean (SD)	Loadings	Mean (SD)	Loadings		Mean (SD)		Loadings	
3. couldn’t experience positive feeling	0.35 (0.62)	0.87	0.53 (0.72)	0.82		0.51 (0.72)		0.84	
5. difficult to work up the initiative to do things	0.35 (0.61)	0.87	0.42 (0.65)	0.78		0.39 (0.65)		0.86	
10. had nothing to look forward to	0.34 (0.63)	0.90	0.47 (0.73)	0.85		0.45 (0.72)		0.88	
13. felt down-hearted and blue	0.39 (0.63)	0.91	0.46 (0.68)	0.91		0.45 (0.69)		0.93	
16. unable to become enthusiastic	0.32 (0.59)	0.91	0.36 (0.64)	0.85		0.36 (0.62)		0.91	
17. not worth much as a person	0.19 (0.50)	0.90	0.15 (0.47)	0.87		0.18 (0.49)		0.88	
21. life was meaningless	0.24 (0.53)	0.89	0.21 (0.52)	0.87		0.23 (0.55)		0.88	
Composite reliability	0.97		0.95				0.96		
Average variance extracted	0.80		0.72				0.78		
Subscale of Anxiety									
2. dryness of my mouth	0.45 (0.66)	0.76	0.78 (0.78)	0.57		0.78 (0.83)		0.66	
4. experienced breathing difficulty	0.31 (0.58)	0.88	0.39 (0.64)	0.82		0.42 (0.69)		0.85	
7. experienced trembling	0.25 (0.56)	0.90	0.23 (0.53)	0.81		0.27 (0.58)		0.84	
9. worried about situations of panic and making a fool of myself	0.47 (0.66)	0.86	0.69 (0.78)	0.78		0.62 (0.76)		0.85	
15. feeling of close to panic	0.30 (0.58)	0.92	0.38 (0.64)	0.91		0.35 (0.63)		0.93	
19. aware of the action of my heart	0.33 (0.59)	0.89	0.38 (0.61)	0.86		0.39 (0.65)		0.86	
20. felt scared without any good reason	0.30 (0.58)	0.91	0.32 (0.61)	0.90		0.32 (0.61)		0.90	
Composite reliability	0.96		0.93				0.95		
Average variance extracted	0.77		0.66				0.71		
Subscale of Stress									
1. hard to wind down	0.48 (0.64)	0.75	0.69 (0.74)		0.60	0.62 (0.72)		0.72	
6. tended to over-react	0.33 (0.60)	0.90	0.40 (0.63)		0.84	0.41 (0.66)		0.85	
8. using a lot of nervous energy	0.48 (0.68)	0.84	0.84 (0.84)		0.70	0.79 (0.84)		0.79	
11. getting agitated	0.36 (0.61)	0.92	0.46 (0.68)		0.92	0.45 (0.69)		0.93	
12. difficult to relax	0.42 (0.65)	0.89	0.54 (0.72)		0.89	0.51 (0.72)		0.90	
14. intolerant of anything	0.43 (0.64)	0.85	0.59 (0.74)		0.71	0.51 (0.68)		0.83	
18. felt rather touchy	0.42 (0.63)	0.85	0.54 (0.71)		0.79	0.53 (0.72)		0.84	
Composite reliability	0.95		0.92				0.94		
Average variance extracted	0.74		0.62				0.70		

Rasch analysis was performed with the purpose of evaluating the item fit of the DASS-21. A Person-Item map was generated (refer to Figure S1 to S3) and it revealed that disordered category thresholds were not present in any of the subscales of the DASS-21 for all samples. Table 5 presents fit statistics showing that all three factors in the DASS-21 were unidimensional in all datasets, including the cross-sectional survey and two waves in the longitudinal survey. This is supported by the following evidence: (i) the eigenvalue ratios (i.e., first eigenvalue / second eigenvalue ratio by PCA) were all higher than 4, and (ii) the eigenvalues of the first component of the residuals were all less than 2. Additionally, there were no substantial misfits among the participants as the standard deviations of the mean person residual were all less than 0.15 (i.e., ranging from − 0.11 to -0.16). Moreover, the person reliability of three subscales was all above 0.70 across all surveys, indicating acceptable internal consistency among school teachers.

**Table 4:** Fit indexes in measurement invariance across different times

Model	χ^2^	*df*	CFI	RMSEA	SRMR	Δχ^2^	Δ*df*	ΔCFI	ΔRMSEA	ΔSRMR
Configural Invariance	2860.18	783	0.996	0.040	0.037	-	-	-	-	-
Thresholds constrained	2915.12	801	0.996	0.040	0.036	54.94	18	0	0	-0.001
Thresholds and loadings constrained	2955.47	819	0.995	0.039	0.042	40.35	18	-0.001	-0.001	0.006
Thresholds, loadings, and intercept constrained	2964.54	822	0.995	0.040	0.042	9.07	3	0	0.001	0
Thresholds, loadings, intercept, and residuals constrained	3014.32	843	0.995	0.039	0.043	49.78	21	0	-0.001	0.001

*Notes*: CFI = comparative fit index; RMSEA = root mean square error of approximation; SRMR = standardized root mean square residual

**Table 5:** Fit statistics of Rasch analysis for the subscales of the DASS-21.

	Cross-sectional survey				Longitudinal survey								
					Wave 1					Wave 2			
	Eigenvalues ratio ^a^	Eigenvalues of residuals	PR	P-residual (SD)	Eigenvalues ratio ^a^	Eigenvalues of residuals	PR	P-residual (SD)	Eigenvalues ratio ^a^		Eigenvalues of residuals	PR	P-residual (SD)
Depression	8.61	1.50	0.78	-0.16 (0.62)	5.69	1.55	0.70	-0.14 (0.69)	6.91		1.59	0.78	− 0.144 (0.65)
Anxiety	7.60	1.59	0.76	-0.15 (0.67)	5.07	1.69	0.70	-0.11 (0.76)	6.13		1.62	0.74	− 0.124 (0.73)
Stress	7.61	1.39	0.77	-0.13 (0.72)	5.18	1.51	0.78	-0.09 (0.82)	7.17		1.47	0.78	− 0.105 (0.78)

^a^ the ratio of first-to-second eigenvalues; PR = person reliability.

Regarding item properties in the Rasch results (Table 6), all items had a point biserial correlation greater than 0.50. This value also indicated good convergent validity. The most difficult items were Item 17 (0.75 logits, “*not worth much as a person*”) in the depression subscale, Item 7 (0.45 logits, “*experienced breathing difficulty*”) in the anxiety subscale, and Item 6 (0.43 logits, “*tended to over-react*”) in the stress subscale. The easiest items were Item 13 (-0.52 logits, “*felt down-hearted and blue*”) in the depression subscale, Item 9 (-0.68 logits, “*worried about situations of panic and making a fool of myself”*) in the anxiety subscale, and Item 8 (-0.37 logits, “*using a lot of nervous energy*”) in the stress subscale. Only a few items were found with unsatisfactory infit and outfit MnSq, including Item 2 (“*dryness of my mouth*”), Item 15 (“*feeling of close to panic*”), and Item 20 (“*felt scared without any good reason*”) in the anxiety subscale; Item 1 (“*hard to wind down*”), Item 11 (“*getting agitated*”), as well as Item 12 (“*difficult to relax*”) in the stress subscale.

**Table 6:** Fit statistics of Rasch model for Items

Source	Cross-sectional survey				Longitudinal survey							
					Wave 1				Wave 2			
	Difficulty	Point-biserial	Infit MnSq	Outfit MnSq	Difficulty	Point-biserial	Infit MnSq	Outfit MnSq	Difficulty	Point-biserial	Infit MnSq	Outfit MnSq
Subscale of Depression												
3. couldn’t experience positive feeling	-0.243	0.801	1.203	1.207	-0.788	0.787	1.149	1.155	-0.727	0.810	1.268	1.277
5. difficult to work up the initiative to do things	-0.130	0.811	1.148	1.133	-0.103	0.741	1.214	1.220	-0.115	0.798	1.136	1.131
10. had nothing to look forward to	-0.309	0.835	0.963	0.952	-0.688	0.792	1.015	1.010	-0.704	0.831	1.011	1.006
13. felt down-hearted and blue	-0.516	0.843	0.990	1.016	-0.558	0.815	0.878	0.873	-0.539	0.844	0.935	0.927
16. unable to become enthusiastic	-0.017	0.843	0.856	0.836	-0.055	0.777	0.879	0.875	0.187	0.838	0.807	0.777
17. not worth much as a person	0.750	0.740	0.890	0.774	1.249	0.609	0.959	0.899	1.159	0.682	0.907	0.526
21. life was meaningless	0.465	0.780	0.910	0.903	0.943	0.689	0.860	0.729	0.739	0.732	0.881	0.954
Subscale of Anxiety												
2. dryness of my mouth	-0.576	0.753	**1.424**	**1.436**	-1.276	0.678	**1.545**	**1.571**	-1.455	0.727	**1.660**	**1.707**
4. experienced breathing difficulty	0.252	0.789	0.921	0.881	0.293	0.730	0.889	0.810	0.049	0.764	0.902	0.849
7. experienced trembling	0.447	0.762	0.828	0.739	0.898	0.635	0.929	0.846	1.056	0.673	0.965	0.809
9. worried about situations of panic and making a fool of myself	-0.680	0.804	1.144	1.175	-1.041	0.756	1.151	1.142	-0.738	0.795	1.059	1.081
15. feeling of close to panic	0.256	0.804	0.816	0.774	0.308	0.749	0.769	0.710	0.407	0.770	**0.692**	**0.581**
19. aware of the action of my heart	0.053	0.801	0.915	0.907	0.326	0.737	0.820	0.823	0.196	0.761	0.859	0.809
20. felt scared without any good reason	0.248	0.799	0.828	0.774	0.492	0.724	**0.735**	**0.598**	0.487	0.743	**0.726**	**0.621**
Subscale of Stress												
1. hard to wind down	-0.125	0.724	**1.450**	**1.419**	-0.144	0.709	**1.446**	**1.459**	-0.144	0.709	**1.446**	**1.459**
6. tended to over-react	0.425	0.774	0.861	0.817	0.605	0.723	0.990	0.947	0.605	0.723	0.947	0.947
8. using a lot of nervous energy	-0.370	0.805	1.027	1.011	-1.059	0.807	1.123	1.138	-1.059	0.807	1.138	1.138
11. getting agitated	0.272	0.807	0.769	0.738	0.312	0.794	**0.682**	**0.607**	0.312	0.794	**0.607**	**0.607**
12. difficult to relax	-0.157	0.831	0.772	0.736	-0.008	0.818	**0.706**	**0.647**	-0.008	0.818	**0.647**	**0.647**
14. intolerant of anything	-0.047	0.791	1.018	0.996	0.295	0.763	1.019	1.019	0.294	0.763	1.019	1.019
18. felt rather touchy	0.002	0.780	1.059	1.050	-0.001	0.770	1.004	0.981	-0.001	0.770	0.981	0.981

Bold means the value was out of the infit and outfit MnSq acceptable range

To visually compare the original and the revised structure of the DASS-21 [(after removing items with unacceptable infit and outfit MnSq (i.e., Item 2, 15, 20, 1, 11, and 12)], network analysis results showed that centrality indices were stable as coefficients were generally above 0.50 (refer to Figure S4-S9). Additionally, the 95% non-parametric CIs for edge weights using the bootstrap method were narrow for the cross-sectional survey dataset, with very few CIs including zero, indicating a high level of accuracy (refer to Figures S10 and S11). However, caution is needed when interpreting the order of these edges in the network for the longitudinal study datasets as the findings were not replicated (refer to Figure S12-S15). Therefore, only the network results obtained from the dataset of the cross-sectional survey are presented.

**Fig. 1:**
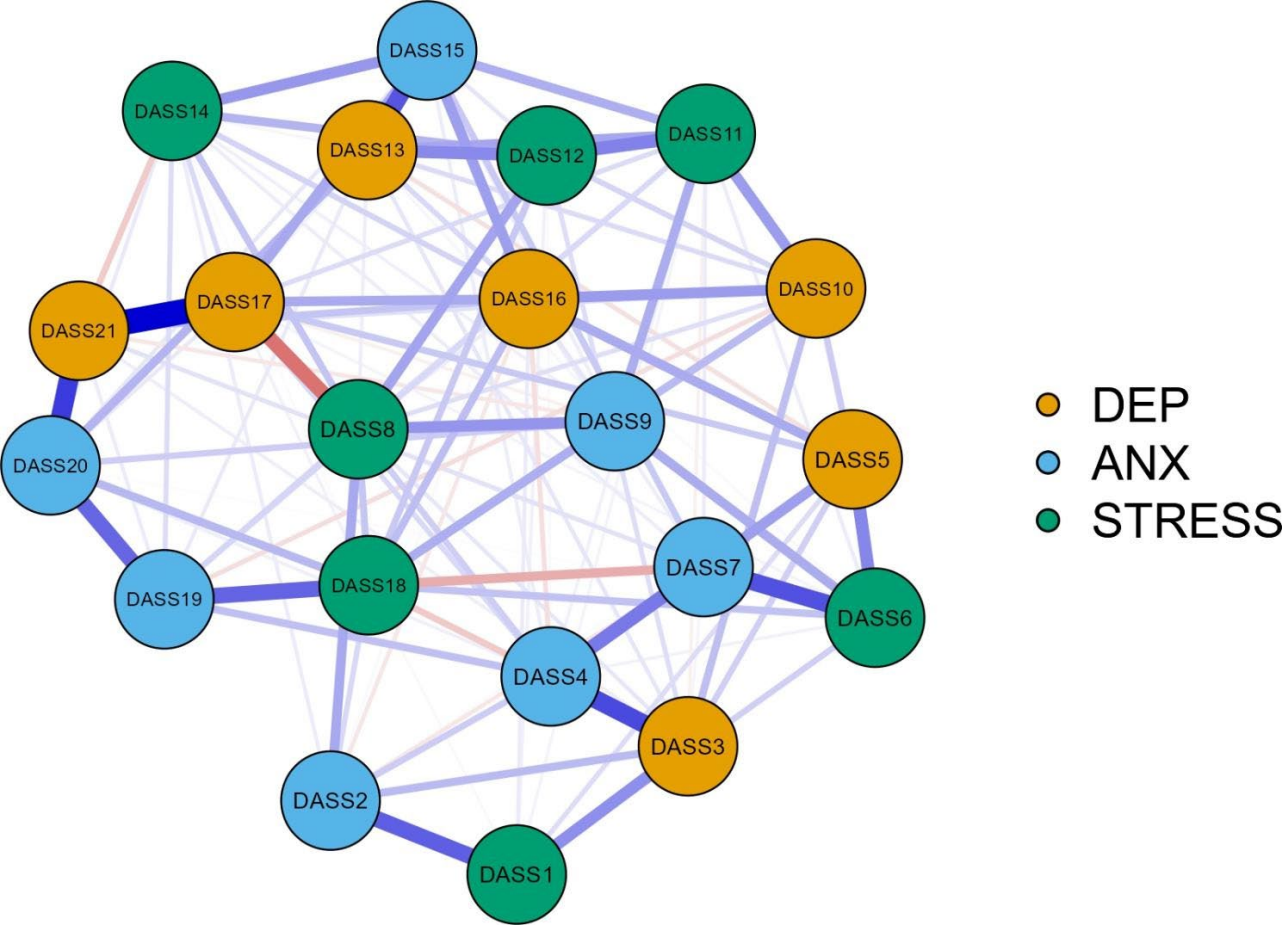
Network analysis of Cross-sectional survey. DEP = Depression; ANX = Anxiety.

**Fig. 2:**
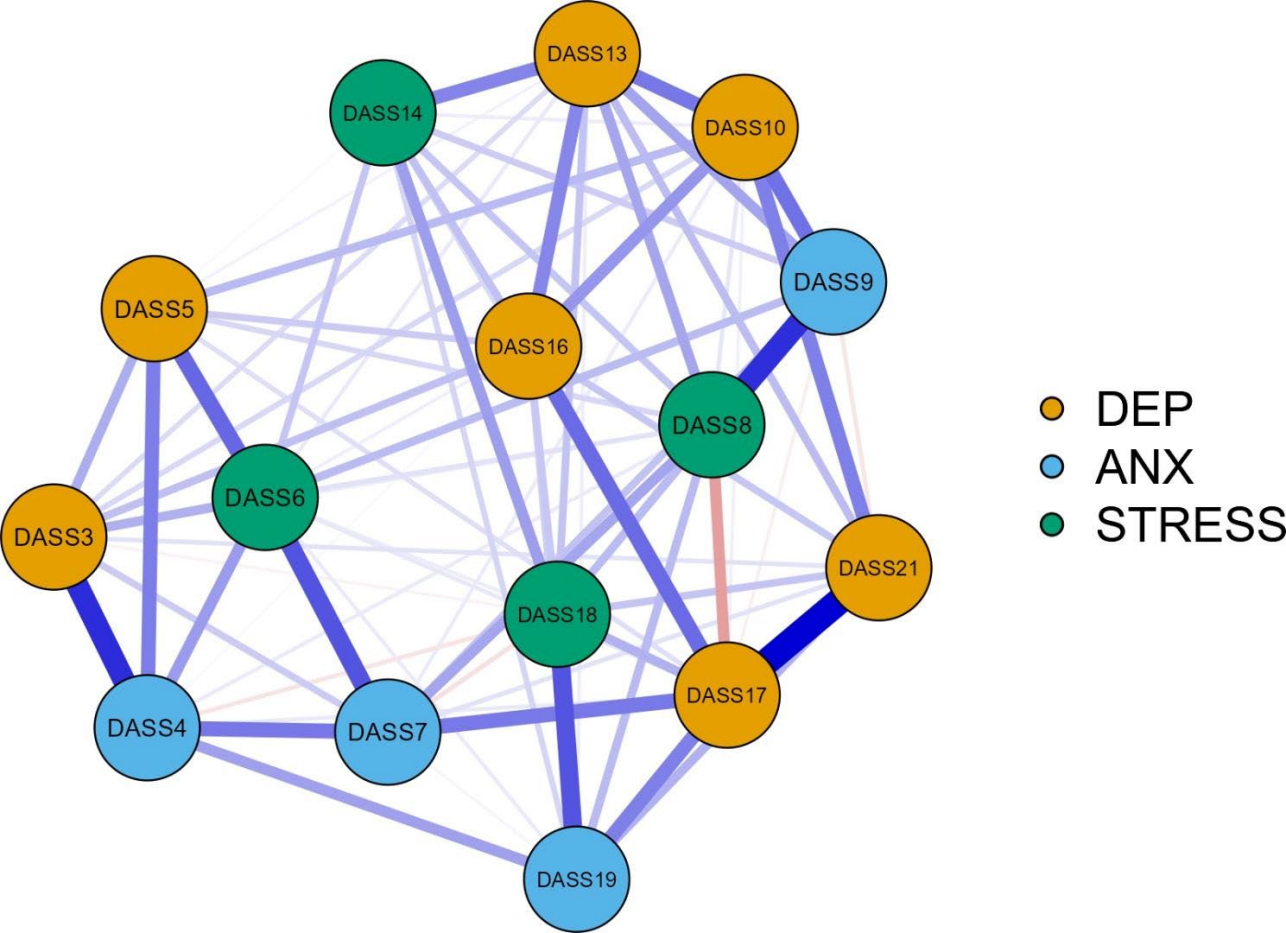
Network analysis of Cross-sectional survey (revised). DEP = Depression; ANX = Anxiety.

The comparison of the DASS-21 networks between the original and revised versions showed that the latter was better aligned with the expected structure (see Figs. 1 and 2). This pattern was consistently found in both waves of the longitudinal study; however, interpretation of edge-weights in these networks should be approached with caution. The centrality indices (see Table S2) revealed higher values in betweenness, closeness, strength, and expected influence for Items 17 (“*not worth much as a person*”) and 13 (“*felt downhearted and blue*”), 7 (“*experienced trembling*”), and 6 (“*tended to overreact*”), indicating that these items had the strongest connections to other nodes (i.e., items).

Table 7 showed that when the inappropriate items retained in the DASS-21, only the factor score of stress at Wave 1 was positively related to emotional exhaustion at Wave 2 (*b* = 4.73, *t* = 7.62, *p* < 0.01), while the other two factors were not. Furthermore, problematic results emerged, that is, depression at Wave 2 and emotional exhaustion at Wave 2 were significantly and negatively correlated (*b* = -1.66, *t* = -2.52, *p* = 0.01). However, stress at Wave 2 was positively related to emotional exhaustion at Wave 2 (*b* = 8.62, *t* = 13.60, *p* < 0.01). Variance inflation factor (VIF) of the original DASS-21 showed that there was a non-negligible multicollinearity problem between explanatory variables and the VIFs of three factors were all higher than 5.50. Alternatively, regression analysis yielded accurate and logical results when inappropriate items were removed. In addition to the unchanged results of the significant positive relationship between stress (including Wave 1 and Wave 2) and emotional exhaustion (*b* at Wave 1 was 3.54, *t* = 6.71, *p* < 0.01; *b* at Wave 2 was 7.48, *t* = 13.95, *p* < 0.01), the relation between depression at Wave 1 and emotional exhaustion was changed to be significantly positive (*b* = 1.24, *t* = 2.13, *p* = 0.03); the original negative association of depression at Wave 2 with emotional exhaustion was also changed to be non-significant. Furthermore, the VIFs of all explanatory variables were under 4.5 after the inappropriate items were removed. Although the values still approximated 5, multicollinearity seemed to be reduced compared to the previously tested version of the instrument.

**Table 7:** Regression model on the emotional exhaustion between original and revised factor score of the DASS-21.

Emotional exhaustion as the dependent variable measured at Wave 2								
	**Wave 1**				**Wave 2**			
	**Original score**: *F* (5, 1596) = 55.95, *p* < 0.01		**Revised score**: *F* (5, 1596) = 49.82, *p* < 0.01		**Original score**: *F* (5, 1596) = 140.67, *p* < 0.01		**Revised score**: *F* (5, 1596) = 138.82, *p* < 0.01	
**Predictor**	**Estimate (SE)**	**Stand. Estimate**	**Estimate (SE)**	**Stand. Estimate**	**Estimate (SE)**	**Stand. Estimate**	**Estimate (SE)**	**Stand. Estimate**
Intercept	21.39 (1.58) ^**^		21.36 (1.59) ^**^		22.78 (1.42) ^**^		23.14 (1.43) ^**^	
Sex	3.51 (0.76) ^**^	0.29	3.50 (0.77) ^**^	0.29	2.92 (0.69) ^**^	0.24	2.71 (0.69) ^**^	0.22
Age	0.10 (0.04) ^**^	0.07	0.10 (0.04) ^**^	0.07	0.08 (0.03) ^*^	0.06	0.07 (0.03) ^*^	0.05
Factor Score - Dep	-0.42 (0.67)	-0.03	1.24 (0.58) *	0.10	-1.66 (0.66) *	-0.13	-0.21 (0.55)	-0.02
Factor Score -Anx	0.54 (0.69)	0.04	0.33 (0.58)	0.02	-0.30 (0.73)	-0.02	-0.06 (0.59)	-0.01
Factor Score - Stress	4.73 (0.62) ^**^	0.37	3.54 (0.53) ^**^	0.26	8.62 (0.63) ^**^	0.68	7.48 (0.54) ^**^	0.56

^**^p < 0.01; ^*^p < 0.05; DEP = Depression; ANX = Anxiety.

## Discussion

Given the escalating levels of stress, depression, and anxiety experienced by teachers—and their subsequent negative impact on both mental and physical health, which may in turn affect student behavior and achievement [8]—the present study evaluated the suitability of the DASS-21 for assessing psychological distress among schoolteachers. The psychometric properties of the DASS-21 were evaluated using three approaches – CFA, Rasch analysis, and network analysis – with the simultaneous aim of providing a comprehensive view. Two surveys, a cross-sectional and a longitudinal survey consisting of two waves with a two-month interval, were employed. The results of the CFA indicated that the DASS-21 demonstrated good factorial validity; the three-factor structure outperformed both the one- and two-factor structures. Additionally, the DASS-21 was found to possess time-invariant properties and excellent convergent validity. However, the scale exhibited relatively poor discriminant validity. The Rasch analysis identified and removed three items with unsatisfactory infit and outfit MnSq from the subscales of anxiety and stress, respectively. Following the removal of these six inappropriate items, the network analysis demonstrated a superior network compared to the original 21-item version. Finally, a regression analysis using emotional exhaustion as the criterion revealed that multicollinearity problems were not found in the revised DASS-21.

### Reliability, factorial, convergent and discriminant validity of the DASS-21

The present study, in line with previous research with teaching staff as samples [32, 33], demonstrated that the DASS-21 had excellent internal consistency in both cross-sectional and longitudinal surveys. Using the CFA approach, we found that all three types of factor structures were acceptable, but compared with one- and two-factor structures, the three-factor structure was much better, which is consistent with previous studies [36, 37, 54]. Moreover, in line with a prior study [72], the test results of the DASS-21’s convergent validity and discriminant validity demonstrated quite different outcomes; the former was found to be excellent, but the latter was poor, indicating that items were highly correlated. Based on these findings, we recommend utilizing the DASS-21 as a composite construct rather than breaking it down into three separate components.

### Time invariance of the DASS-21

The DASS-21 has been previously investigated for measurement invariance across various factors such as cultures [36] and gender [28]. However, to the best of our knowledge, no study has examined the time invariance of the DASS-21 in a longitudinal study of teachers’ population. In the current study, the three-factor structure of this instrument was found to remain invariant across a two-month interval, suggesting that the instrument had consistent construct and item descriptions over time. This finding provides solid evidence for mental health interventions aimed at teachers experiencing psychological distress, as only a time-invariant instrument allows for the evaluation of the same concepts over time [73]. This property enables healthcare providers and researchers to compare the levels of teachers’ psychological distress before and after interventions, and assess their effectiveness.

### Three approaches of psychometric evaluation of the DASS-21

To our knowledge, the current study is the first to combine CFA, Rasch analysis as well as network analysis to evaluate the properties of the DASS-21. Using Rasch analysis to scrutinize the properties of the individual items of the DASS-21, the present study found 6 unsatisfactory items, with three items (Items 2, 15, and 20) from the anxiety subscale, three (Items 1, 11, and 12) from the stress subscale, and none from the depression subscale. This finding differs from Shea et al.’s [38] and Medvedev et al.’s [39] studies applying Rasch analysis to the DASS-21. In order to achieve the best model fit, the former study removed Item 5 (depression subscale), Item 2 (anxiety subscale), and Item 11 (stress subscale), while the latter only removed Item 5 from the depression subscale. Subsequently, by visually comparing the original 21-item version with the revised 15-item version using network analysis, we demonstrated that revisions to the DASS structure were consistent with the original version. Furthermore, using emotional exhaustion as a criterion, we found that the revised version had good criterion validity through regression analysis. On account of this, we recommend using CFA, Rasch analysis, and network analysis in conjunction, as they complement each other. For example, network analysis can help to visually illustrate the changes that occur after the removal of inappropriate items. The combination of these three analyses is proposed to be applied to the evaluation of widely used scales with disputes such as SCL-90 and SCL-90-R.

### Strengths and limitation

The strengths of this study include the large sample size and the multiple methods to assess the psychometric properties for the DASS-21 among the teacher population. This study provides several key advancements in the understanding and application of the scale. In line with findings from previous studies conducted on different populations [27–31], our study reaffirms the three-factor structure of the scale. By confirming its validity in our population, we are providing further robustness to the DASS-21’s application for both research and clinical use on teachers. A particularly significant contribution is the identification of DASS-21’s time invariance, an unexplored attribute that facilitates longitudinal assessments of psychological distress, critical for measuring the effectiveness of mental health interventions. The study introduces an innovative methodological combination of CFA, Rasch analysis, and network analysis for a more comprehensive evaluation. In contrast to studies that are limited to integrating a maximum of two methods [74, 75], our approach unveils potential areas of refinement, leading to the proposition of an optimized 15-item version. Additionally, this methodology establishes a foundation for evaluating other contested psychological scales. Essentially, this study enhances DASS-21’s theoretical comprehension and practical use.

Despite the contributions listed above, it is important to note there are some limitations to the present study. First, the DASS-21’s criterion validity was evaluated by assessing participants’ emotional exhaustion only at Wave 2 of the longitudinal study. Therefore, the relevance of the data for participants in the cross-sectional survey and Wave 1 of the longitudinal survey remains unclear. Second, non-probability sampling was used in the study, so results may be limited in their generalizability, despite the large cross-sectional sample.

## Conclusions

In summary, this study affirms the DASS-21 as a reliable, valid, and time-invariant tool for assessing mental health among teachers in primary and middle schools. The combined use of CFA, Rasch analysis, and network analysis offers an innovative method for evaluating psychometric properties, emphasizing excellent convergent validity but poor discriminant validity. Despite confirming the superiority of the three-factor structure, the study suggests that it is more beneficial to use the DASS-21 as a measure of overall psychological distress rather than treating depression, anxiety, and stress as distinct constructs. This research paves the way for future studies to employ similar methodological combinations in evaluating widely disputed psychological instruments.

## Electronic supplementary material

Below is the link to the electronic supplementary material.

Supplementary Material 1

## Data Availability

The datasets used and/or analyzed during the current study are available from the corresponding author on reasonable request.

## Abbreviations

DASS-21: Depression, Anxiety, and Stress scale-21
CFA: Confirmatory factor analysis
SSO: Stressor?Strain?Outcome
IRB: Institutional Review Board
CTJBO: Chinese Teachers? Job Burnout Questionnaire
MBI: Maslach Burnout Inventory
CFI: Comparative fit index
NNFI: Non-normed fit index
AIC: Akaike information criterion
CR: Composite reliability
AVE: Average variance extracted
SRMR: Standardized root mean square residual
RSM: Andrich Rating Scale Model
PCM: Partial Credit Rasch Model
PCA: principal component analysis
Infit: Information-weighted fit statistic
MnSq: Mean square
EBICglasso: Extended Bayesian Information Criterion Graphical Least Absolute Shrinkage and Selection Operator
CIs: Confidence intervals
SCL-90: Symptom check list 90
SCL-90-R: Symptom check list 90 revised
NAA: Naphtha acetic acid

## References

[1] Ramberg J, Låftman SB, Nilbrink J, Olsson G, Toivanen S (2022). Job strain and sense of coherence: Associations with stress-related outcomes among teachers. Scand J Public Health.

[2] Carroll A, York A, Fynes-Clinton S, Sanders-O’Connor E, Flynn L, Bower JM, Forrest K, Ziaei M (2021). The downstream effects of teacher well-being programs: improvements in teachers’ stress, cognition and well-being benefit their students. Front Psychol.

[3] Titheradge D, Hayes R, Longdon B, Allen K, Price A, Hansford L, Nye E, Ukoumunne OC, Byford S, Norwich B (2019). Psychological distress among primary school teachers: a comparison with clinical and population samples. Public Health.

[4] Chirico F (2017). Religious belief and mental health in lay and consecrated italian teachers. J Relig Health.

[5] Martínez-Monteagudo MC, Inglés CJ, Granados L, Aparisi D, García-Fernández JM (2019). Trait emotional intelligence profiles, burnout, anxiety, depression, and stress in secondary education teachers. Pers Indiv Differ.

[6] Stapleton P, Garby S, Sabot D (2020). Psychological distress and coping styles in teachers: a preliminary study. Australian J Educ.

[7] Fernández-Batanero J-M, Román-Graván P, Reyes-Rebollo M-M, Montenegro-Rueda M (2021). Impact of educational technology on teacher stress and anxiety: a literature review. Int J Environ Res Public Health.

[8] Chen I-H, Chen C-Y, Zhao K-Y, Gamble JH, Lin C-Y, Griffiths MD, Pakpour AH. Psychometric evaluation of fear of COVID-19 scale (FCV-19S) among chinese primary and middle schoolteachers, and their students. Curr Psychol 2022:1–17. 10.1007/s12144-021-02471-3 PMC8727075 35002189

[9] Collie RJ, Mansfield CF (2022). Teacher and school stress profiles: a multilevel examination and associations with work-related outcomes. Teach Teacher Educ.

[10] García-Carmona M, Marín MD, Aguayo R (2019). Burnout syndrome in secondary school teachers: a systematic review and meta-analysis. Soc Psychol Educ.

[11] Desouky D, Allam H (2017). Occupational stress, anxiety and depression among egyptian teachers. J Epidemiol global health.

[12] Skaalvik EM, Skaalvik S (2010). Teacher self-efficacy and teacher burnout: a study of relations. Teach teacher Educ.

[13] Phillips AC, Carroll D, Der G (2015). Negative life events and symptoms of depression and anxiety: stress causation and/or stress generation. Anxiety Stress & Coping.

[14] Montgomery C, Rupp AA. A meta-analysis for exploring the diverse causes and effects of stress in teachers. Can J Education/Revue canadienne de l’éducation 2005:458–86.

[15] Richards R, Hemphill KA, Templin MA (2018). Personal and contextual factors related to teachers’ experience with stress and burnout. Teachers and Teaching.

[16] Kim LE, Jörg V, Klassen RM (2019). A meta-analysis of the effects of teacher personality on teacher effectiveness and burnout. Educational Psychol Rev.

[17] von der Embse N, Ryan SV, Gibbs T, Mankin A (2019). Teacher stress interventions: a systematic review. Psychol Sch.

[18] Yang R, You X, Zhang Y, Lian L, Feng W (2019). Teachers’ mental health becoming worse: the case of China. Int J Educational Dev.

[19] Carrozzino D, Siri C, Bech P (2019). The prevalence of psychological distress in Parkinson’s disease patients: the brief symptom inventory (BSI-18) versus the Hopkins symptom checklist (SCL-90-R). Prog Neuropsychopharmacol Biol Psychiatry.

[20] Lovibond SH, Lovibond PF (1996). Manual for the depression anxiety stress scales.

[21] Gong X, Xie X-y, Xu R, Luo Y-j (2010). Psychometric properties of the chinese versions of DASS-21 in chinese college students. Chin J Clin Psychol.

[22] Ali AM, Green J (2019). Factor structure of the depression anxiety stress Scale-21 (DASS-21): Unidimensionality of the arabic version among egyptian drug users. Subst Abuse Treat Prev Policy.

[23] Ahmed O, Faisal RA, Alim SMAHM, Sharker T, Hiramoni FA (2022). The psychometric properties of the depression anxiety stress scale-21 (DASS-21) Bangla version. Acta Psychol.

[24] Kumar K, Kumar S, Mehrotra D, Tiwari SC, Kumar V, Dwivedi RC (2019). Reliability and psychometric validity of Hindi version of depression, anxiety and stress scale-21 (DASS-21) for Hindi speaking head neck cancer and oral potentially malignant disorders patients. J Cancer Res Ther.

[25] Thiyagarajan A, James TG, Marzo RR (2022). Psychometric properties of the 21-item depression, anxiety, and stress scale (DASS-21) among Malaysians during COVID-19: a methodological study. Humanit Social Sci Commun.

[26] Kakemam E, Navvabi E, Albelbeisi AH, Saeedikia F, Rouhi A, Majidi S (2022). Psychometric properties of the Persian version of depression anxiety stress Scale-21 items (DASS-21) in a sample of health professionals: a cross-sectional study. BMC Health Serv Res.

[27] Lee B, Kim YE (2022). Validity of the depression, anxiety, and stress scale (DASS-21) in a sample of korean university students. Curr Psychol.

[28] Vaughan RS, Edwards EJ, MacIntyre TE (2020). Mental health measurement in a post Covid-19 world: psychometric properties and invariance of the DASS-21 in athletes and non-athletes. Front Psychol.

[29] Evans L, Haeberlein K, Chang A, Handal P (2021). Convergent validity and preliminary cut-off scores for the anxiety and depression subscales of the DASS-21 in US adolescents. Child Psychiatry & Human Development.

[30] Reilly C, Atkinson P, Memon A, Jones C, Dabydeen L, Das KB, Gillberg C, Neville BG, Scott RC (2018). Symptoms of depression, anxiety, and stress in parents of young children with epilepsy: a case controlled population-based study. Epilepsy Behav.

[31] Belcher J, Wuthrich VM, Lowe C (2022). Teachers use of fear appeals: Association with student and teacher mental health. Br J Educ Psychol.

[32] Cooper CM, Przeworski A, Smith AC, Obeid R, Short EJ. Perceptions of social–emotional learning among K-12 Teachers in the USA during the COVID-19 pandemic. School Mental Health 2023:1–14. 10.1007/s12310-022-09563-w PMC9838265 36686286

[33] Kukreti S, Ahorsu DK, Strong C, Chen I-H, Lin C-Y, Ko N-Y, Griffiths MD, Chen Y-P, Kuo Y-J, Pakpour AH (2021). Post-traumatic stress disorder in chinese teachers during COVID-19 pandemic: roles of fear of COVID-19, nomophobia, and psychological distress. Healthcare.

[34] Tran TD, Tran T, Fisher J (2013). Validation of the depression anxiety stress scales (DASS) 21 as a screening instrument for depression and anxiety in a rural community-based cohort of northern vietnamese women. BMC Psychiatry.

[35] Lee J, Lee E-H, Moon SH (2019). Systematic review of the measurement properties of the Depression anxiety stress Scales–21 by applying updated COSMIN methodology. Qual Life Res.

[36] Bibi A, Lin M, Zhang XC, Margraf J (2020). Psychometric properties and measurement invariance of Depression, anxiety and stress scales (DASS-21) across cultures. Int J Psychol.

[37] Gomez R, Stavropoulos V, Griffiths MD (2020). Confirmatory factor analysis and exploratory structural equation modelling of the factor structure of the Depression anxiety and stress Scales-21. PLoS ONE.

[38] Tennant A, Conaghan PG (2007). The Rasch measurement model in rheumatology: what is it and why use it? When should it be applied, and what should one look for in a Rasch paper?. Arthritis Care Res.

[39] Wongpakaran N, Wongpakaran T, Pinyopornpanish M, Simcharoen S, Suradom C, Varnado P, Kuntawong P (2020). Development and validation of a 6-item revised UCLA Loneliness Scale (RULS‐6) using rasch analysis. Br J Health Psychol.

[40] Sbeglia GC, Nehm RH (2019). Do you see what I-SEA? A rasch analysis of the psychometric properties of the Inventory of Student Evolution Acceptance. Sci Educ.

[41] Boone WJ (2016). Rasch analysis for instrument development: why, when, and how?. CBE—Life Sci Educ.

[42] van der Wal MB, Tuinebreijer WE, Bloemen MC, Verhaegen PD, Middelkoop E, van Zuijlen PP (2012). Rasch analysis of the patient and Observer Scar Assessment Scale (POSAS) in burn scars. Qual Life Res.

[43] Shea TL, Tennant A, Pallant JF (2009). Rasch model analysis of the Depression, anxiety and stress scales (DASS). BMC Psychiatry.

[44] Medvedev ON, Krägeloh CU, Titkova EA, Siegert RJ (2020). Rasch analysis and ordinal-to-interval conversion tables for the Depression, anxiety and stress scale. J Health Psychol.

[45] Cai Y, Dong S, Yuan S, Hu C-P (2020). Network analysis and its applications in psychology. Adv Psychol Sci.

[46] Beard C, Millner AJ, Forgeard MJ, Fried EI, Hsu KJ, Treadway MT, Leonard CV, Kertz S, Björgvinsson T (2016). Network analysis of depression and anxiety symptom relationships in a psychiatric sample. Psychol Med.

[47] Borsboom D, Cramer AO (2013). Network analysis: an integrative approach to the structure of psychopathology. Ann Rev Clin Psychol.

[48] Van den Bergh N, Marchetti I, Koster EH (2021). Bridges over troubled waters: mapping the interplay between anxiety, depression and stress through network analysis of the DASS-21. Cogn Therapy Res.

[49] Van den Bergh N, Marchetti I, Koster EH. Bridges over troubled waters: mapping the interplay between anxiety, depression and stress through network analysis of the DASS-21. Cogn Therapy and Res. 2021;45:46–60.

[50] Koeske GF, Koeske RD (1993). A preliminary test of a stress-strain-outcome model for reconceptualizing the burnout phenomenon. J Social Service Res.

[51] Ozoemena EL, Agbaje OS, Ogundu L, Ononuju AH, Umoke PCI, Iweama CN, Kato GU, Isabu AC, Obute AJ (2021). Psychological distress, burnout, and coping strategies among nigerian primary school teachers: a school-based cross-sectional study. BMC Public Health.

[52] Yin H, Huang S, Chen G (2019). The relationships between teachers’ emotional labor and their burnout and satisfaction: a meta-analytic review. Educational Res Rev.

[53] Chen I-H, Chen X-M, Liao X-L, Zhao K-Y, Wei Z-H, Lin C-Y, Gamble JH (2022). Evaluating the immediate and delayed effects of psychological need thwarting of online teaching on chinese primary and middle school teachers’ psychological well-being. Front Psychol.

[54] Cao C-H, Dang C-Y, Zheng X, Chen W-G, Chen I-H, Gamble JH (2023). The Psychometric Properties of the DASS-21 and its Association with Problematic Internet Use among Chinese College Freshmen. Healthcare.

[55] Wu X-c, Qi Y-j, Yu R-r (2016). Zang W-w: revision of chinese primary and secondary school teachers’ job burnout questionnaire. Chin J Clin Psychol.

[56] Maslach C, Jackson SE (1981). The measurement of experienced burnout. J organizational Behav.

[57] Szabó M, Lovibond PF (2006). Anxiety, depression, and tension/stress in children. J Psychopathol Behav Assess.

[58] Hu Lt, Bentler PM (1999). Cutoff criteria for fit indexes in covariance structure analysis: conventional criteria versus new alternatives. Struct equation modeling: multidisciplinary J.

[59] Fränzi Korner-Nievergelt TR. Stefanie von Felten, Jérôme Guélat, Bettina Almasi, Korner-Nievergelt: Model Selection and Multimodel Inference. In: *Bayesian Data Analysis in Ecology Using Linear Models with R, BUGS, and STAN* edn. Edited by Fränzi Korner-Nievergelt TR, Stefanie von Felten, Jérôme Guélat, Bettina Almasi, Korner-Nievergelt: Academic Press; 2015:175–196.

[60] Joseph F, Hair BJB, Rolph E, Anderson WC, Black. Multivariate Data Analysis. 8th ed. Cengage Learning EMEA; 2018.

[61] Wu H, Estabrook R (2016). Identification of confirmatory factor analysis dodels of different levels of invariance for ordered categorical outcomes. Psychometrika.

[62] Chen FF (2007). Sensitivity of goodness of fit indexes to lack of measurement invariance. Struct equation modeling: multidisciplinary J.

[63] Slocum-Gori SL, Zumbo BD (2011). Assessing the unidimensionality of psychological scales: using multiple criteria from factor analysis. Soc Indic Res.

[64] Linacre J. Rasch measurement computer program user’s guide. Beaverton Oregon: Winsteps 2016.

[65] Morris RL, Soh S-E, Hill KD, Buchbinder R, Lowthian JA, Redfern J, Etherton-Beer CD, Hill A-M, Osborne RH, Arendts G (2017). Measurement properties of the health literacy questionnaire (HLQ) among older adults who present to the emergency department after a fall: a Rasch analysis. BMC Health Serv Res.

[66] Anderson DL, Fisher KM, Norman GJ (2002). Development and evaluation of the conceptual inventory of natural selection. J Res Sci Teach.

[67] Kaplan RM, Saccuzzo DP. Psychological testing: principles, applications, and issues. Cengage Learning; 2017.

[68] Tang WK, Wong E, Chiu HF, Lum C, Ungvari GS (2005). The geriatric Depression Scale should be shortened: results of Rasch analysis. Int J Geriatr Psychiatry.

[69] Epskamp S, Cramer AO, Waldorp LJ, Schmittmann VD, Borsboom D (2012). qgraph: Network visualizations of relationships in psychometric data. J Stat Softw.

[70] Robinaugh DJ, Millner AJ, McNally RJ (2016). Identifying highly influential nodes in the complicated grief network. J Abnorm Psychol.

[71] Epskamp S, Borsboom D, Fried EI (2018). Estimating psychological networks and their accuracy: a tutorial paper. Behav Res Methods.

[72] Lee D (2019). The convergent, discriminant, and nomological validity of the Depression anxiety stress Scales-21 (DASS-21). J Affect Disord.

[73] Chen I-H, Strong C, Lin Y-C, Tsai M-C, Leung H, Lin C-Y, Pakpour AH, Griffiths MD (2020). Time invariance of three ultra-brief internet-related instruments: Smartphone application-based addiction scale (SABAS), Bergen social media addiction scale (BSMAS), and the nine-item internet gaming disorder scale-short form (IGDS-SF9)(study part B). Addict Behav.

[74] Bilbao A, Martín-Fernández J, García-Pérez L, Mendezona JI, Arrasate M, Candela R, Acosta FJ, Estebanez S, Retolaza A (2022). Psychometric properties of the EQ-5D-5L in patients with major depression: factor analysis and rasch analysis. J Mental Health.

[75] Baek JJH, Soares GH, da Rosa GC, Mialhe FL, Biazevic MGH, Michel-Crosato E (2021). Network analysis and psychometric properties of the brazilian version of the eHealth literacy scale in a dental clinic setting. Int J Med Informatics.

