# Supplementary material for: Psychometric evaluation of the depression, anxiety, and stress scale-21 (DASS-21) among Chinese primary and middle school teachers
Source: BMC Psychol. 2023 Jul 14;11:209. doi: 10.1186/s40359-023-01242-y (PMC10349442; doi:10.1186/s40359-023-01242-y)
Supplement: Supplementary file 1 — Supplementary Material 1 [file 40359_2023_1242_MOESM1_ESM.docx]

**Table S1.** The test of the discriminant validity among the subscale of the DASS-21.

|  | Depression | Anxiety | Stress |
| --- | --- | --- | --- |
| Depression | **.89/.84/.88** |  |  |
| Anxiety | .99/.99/.99 | **.88/.81/.84** |  |
| Stress | .98/.96/.96 | .99/.97/.99 | **.86/.79/.84** |

*Notes:* Data source from left to right: cross-sectional survey, longitudinal survey (wave 1), longitudinal survey (wave 2). Diagonal elements in bold are square root of averaged variance extracted. When these values were higher than the inter-latent factors correlations (off-diagonal elements), the discriminant validity was support for the respective latent variable.

**Table S2.** Centrality measures of cross-sectional survey.

|  | | Network | | | | | | | |
| --- | --- | --- | --- | --- | --- | --- | --- | --- | --- |
| Variable | | Betweenness | | Closeness | | Strength | | Expected influence | |
| DASS3 |  | -0.735 |  | -1.503 |  | -1.074 |  | -1.070 |  |
| DASS5 |  | -0.735 |  | -0.897 |  | -1.076 |  | -0.740 |  |
| DASS10 |  | -0.086 |  | 0.391 |  | -0.349 |  | 0.297 |  |
| DASS13 |  | -0.519 |  | -0.565 |  | 0.438 |  | 1.418 |  |
| DASS16 |  | -0.303 |  | 0.717 |  | 0.076 |  | 0.903 |  |
| DASS17 |  | 2.508 |  | 2.140 |  | 2.618 |  | 1.255 |  |
| DASS21 |  | -0.519 |  | 0.525 |  | -0.028 |  | 0.029 |  |
| DASS4 |  | 0.778 |  | -0.258 |  | 0.194 |  | 0.320 |  |
| DASS7 |  | 1.859 |  | 1.164 |  | 0.555 |  | 0.711 |  |
| DASS9 |  | -0.735 |  | -0.085 |  | 0.002 |  | -0.362 |  |
| DASS19 |  | -0.303 |  | 0.534 |  | -0.490 |  | 0.095 |  |
| DASS6 |  | 0.346 |  | -0.193 |  | 0.257 |  | 1.160 |  |
| DASS8 |  | -0.086 |  | 0.418 |  | 0.733 |  | -0.997 |  |
| DASS14 |  | -0.951 |  | -1.594 |  | -1.824 |  | -1.806 |  |
| DASS18 |  | -0.519 |  | -0.793 |  | -0.034 |  | -1.213 |  |
|  | | | | | | | | | |

| 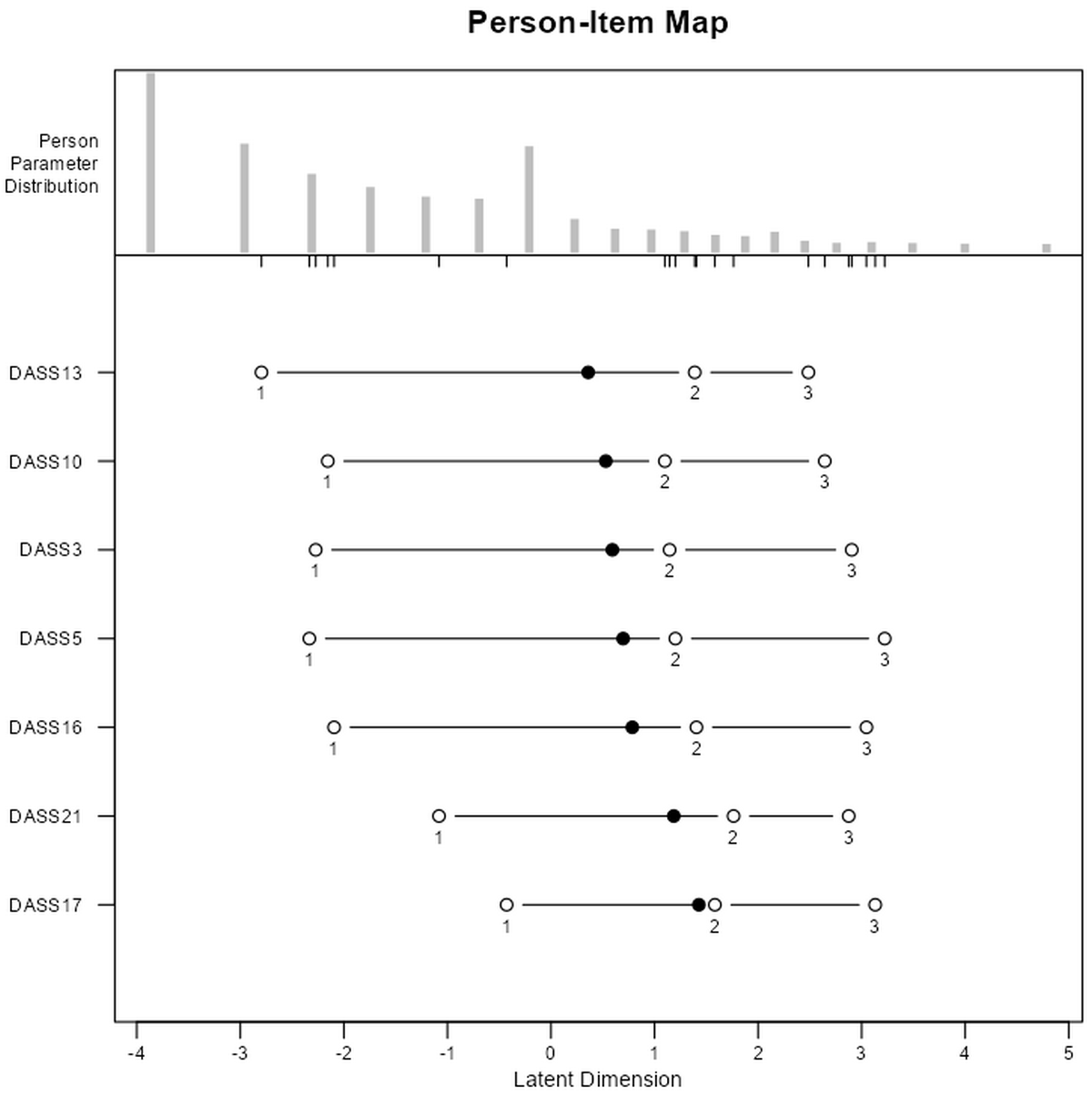 | 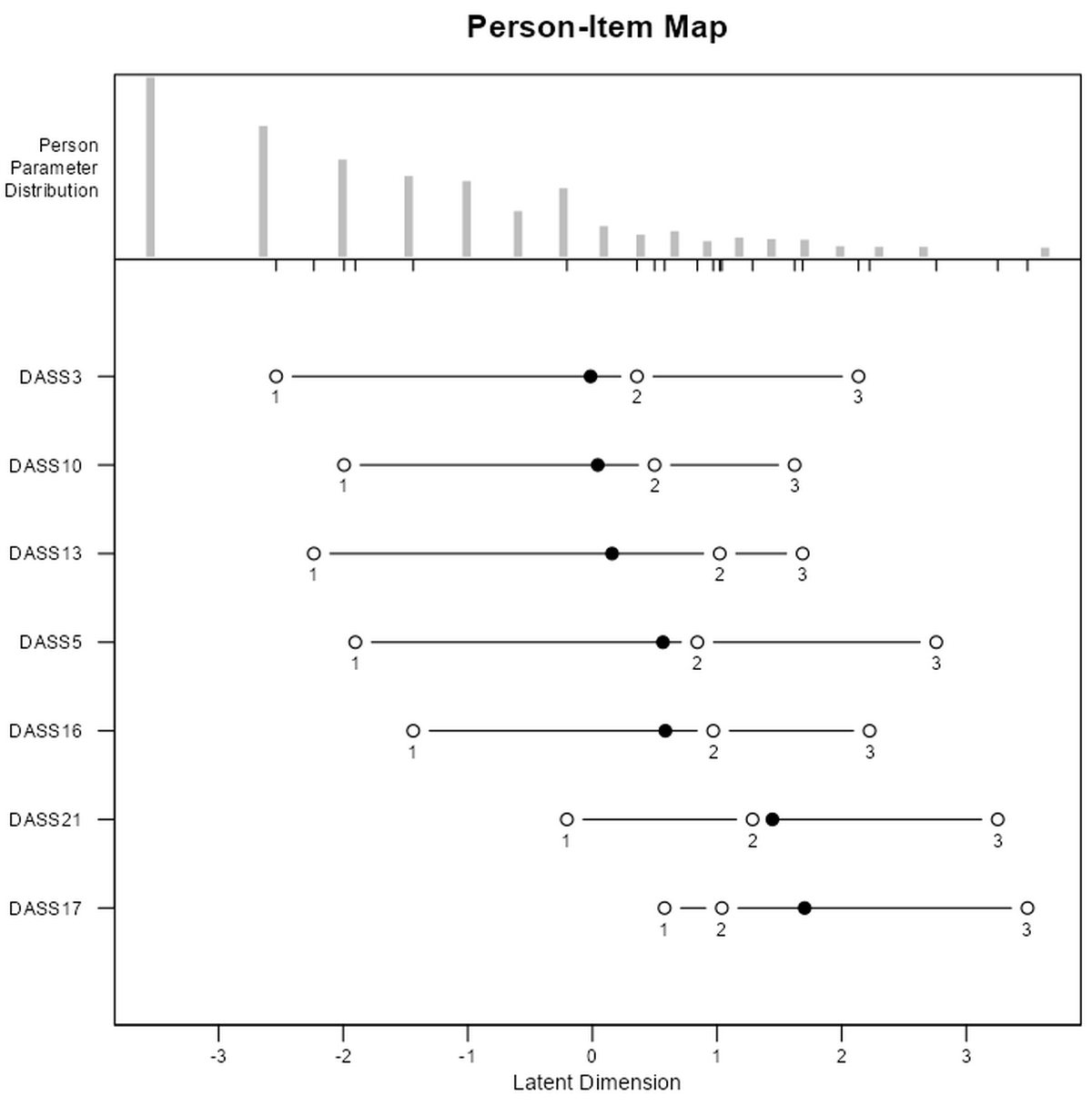 | 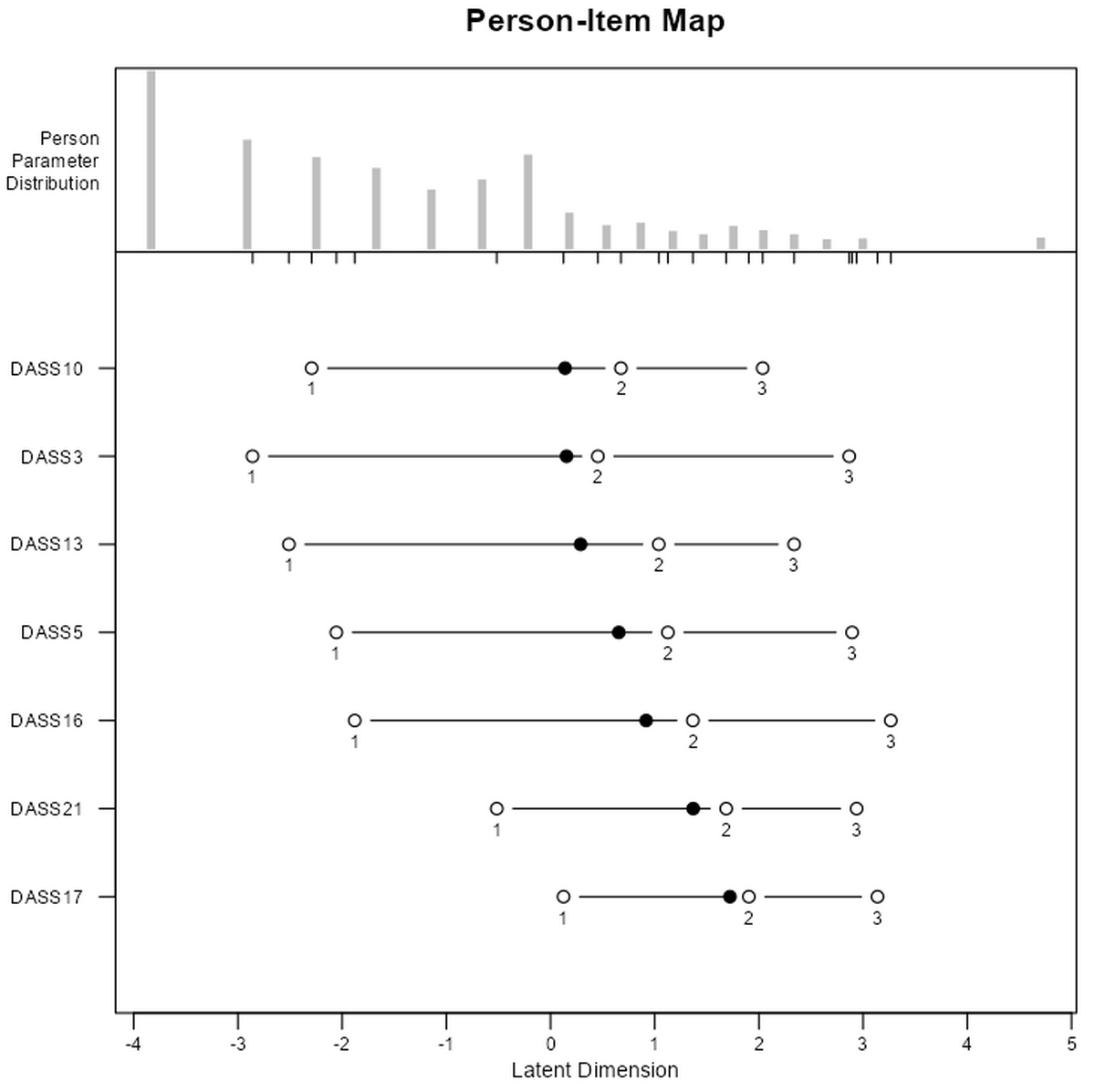 |
| --- | --- | --- |
| 2020 | 2021 | 2022 |

**Figure S1.** Person-Item map of the depression subscale.

| 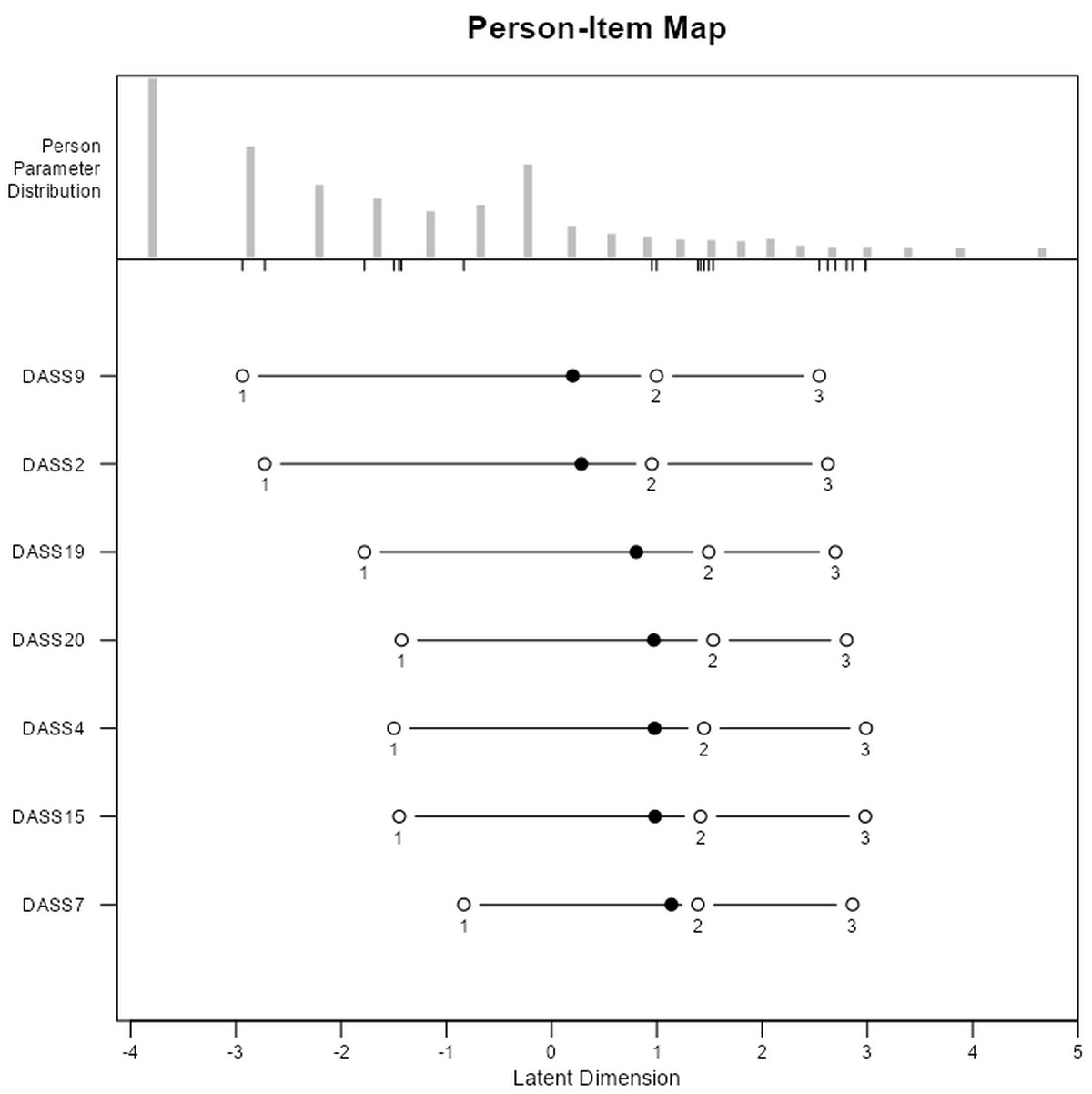 | 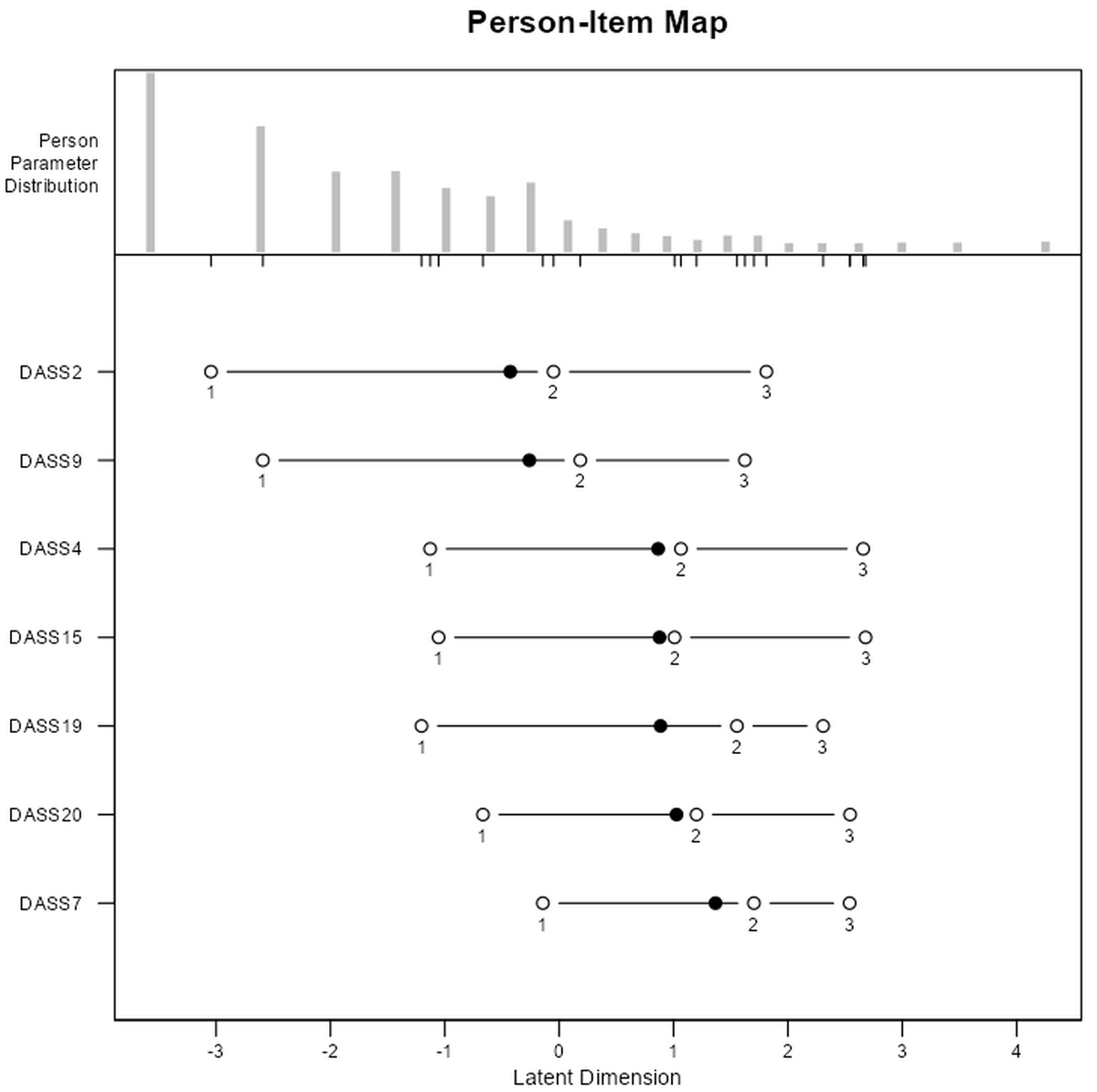 | 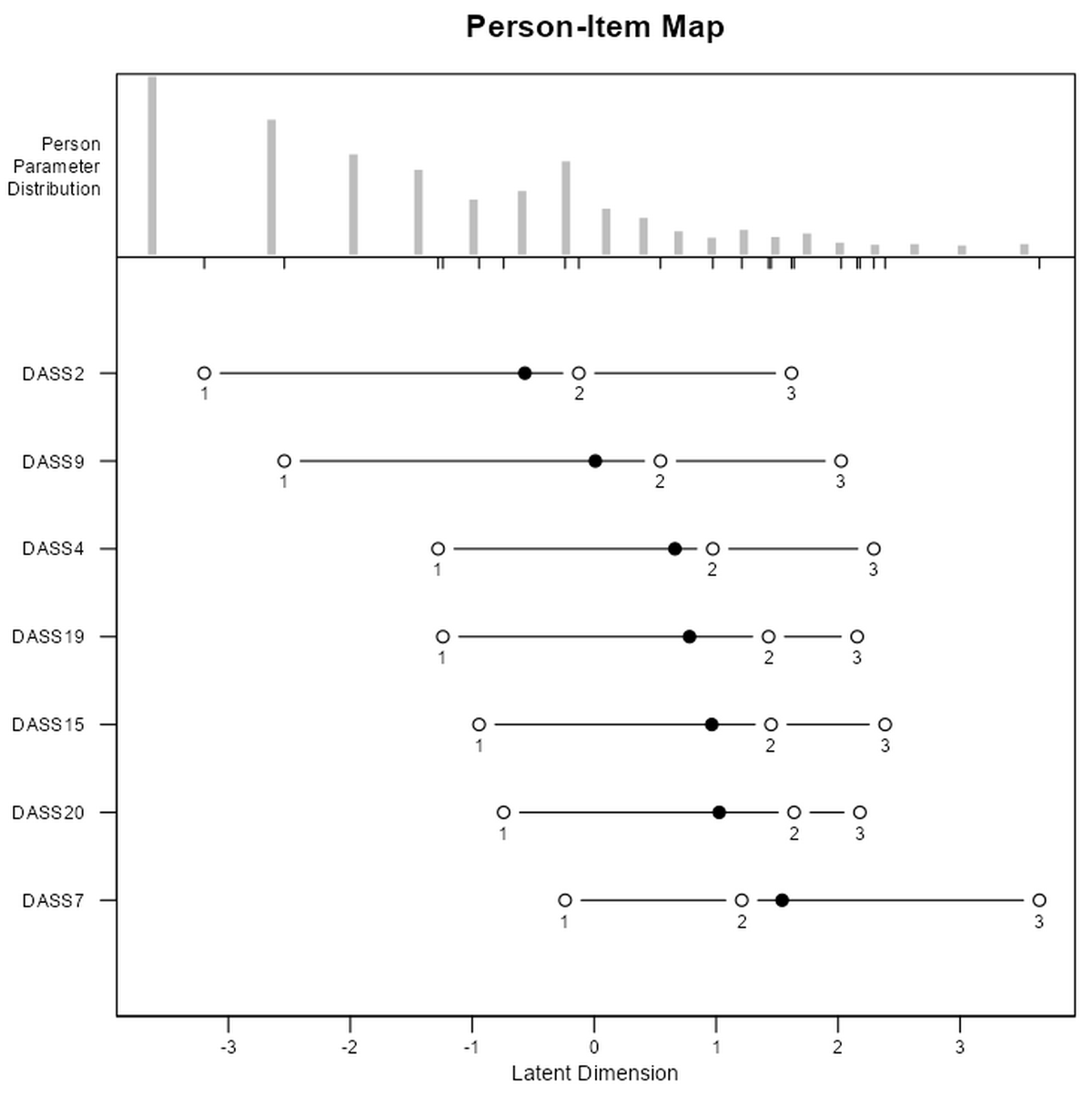 |
| --- | --- | --- |
| 2020 | 2021 | 2022 |

| 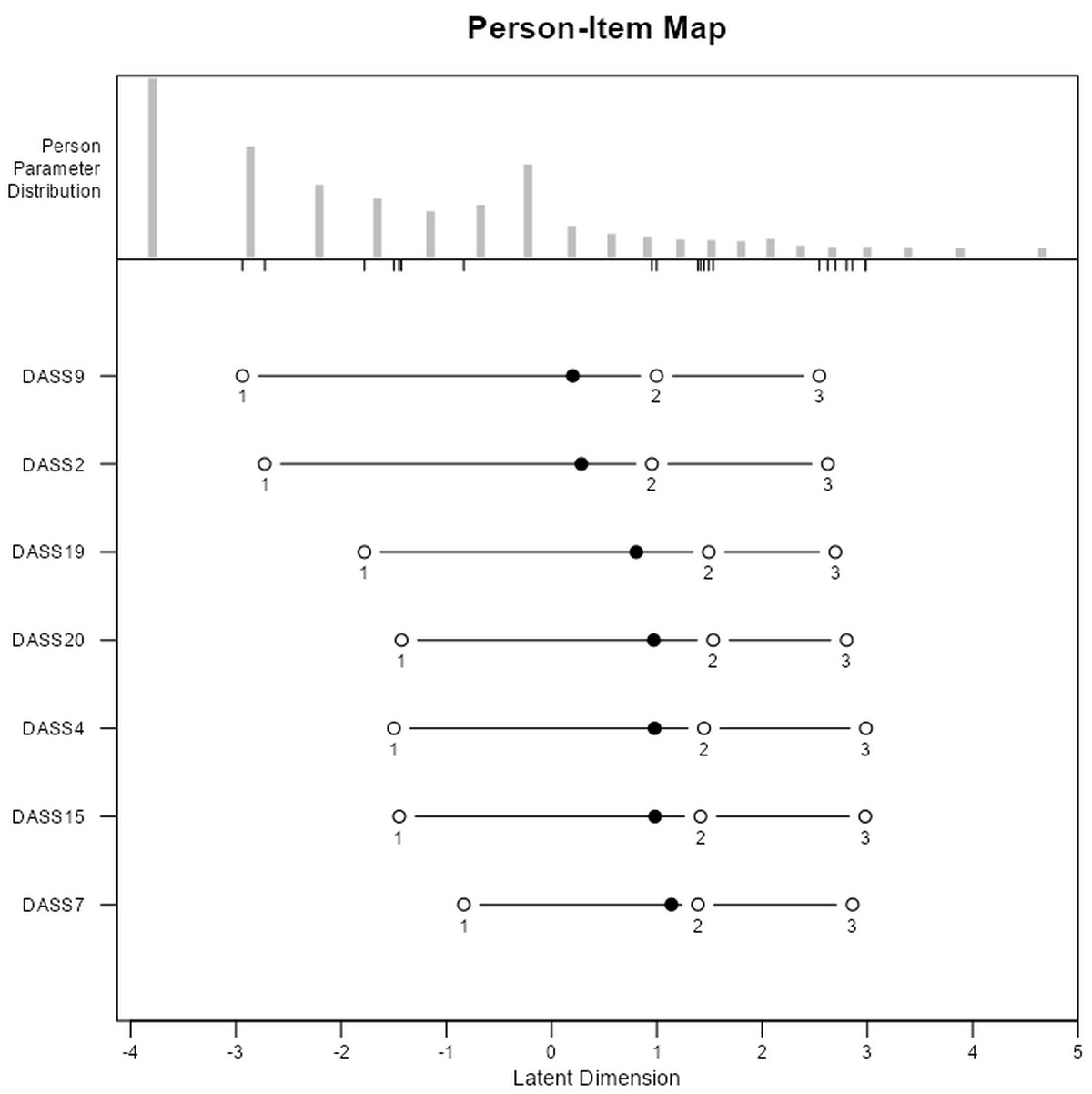 | 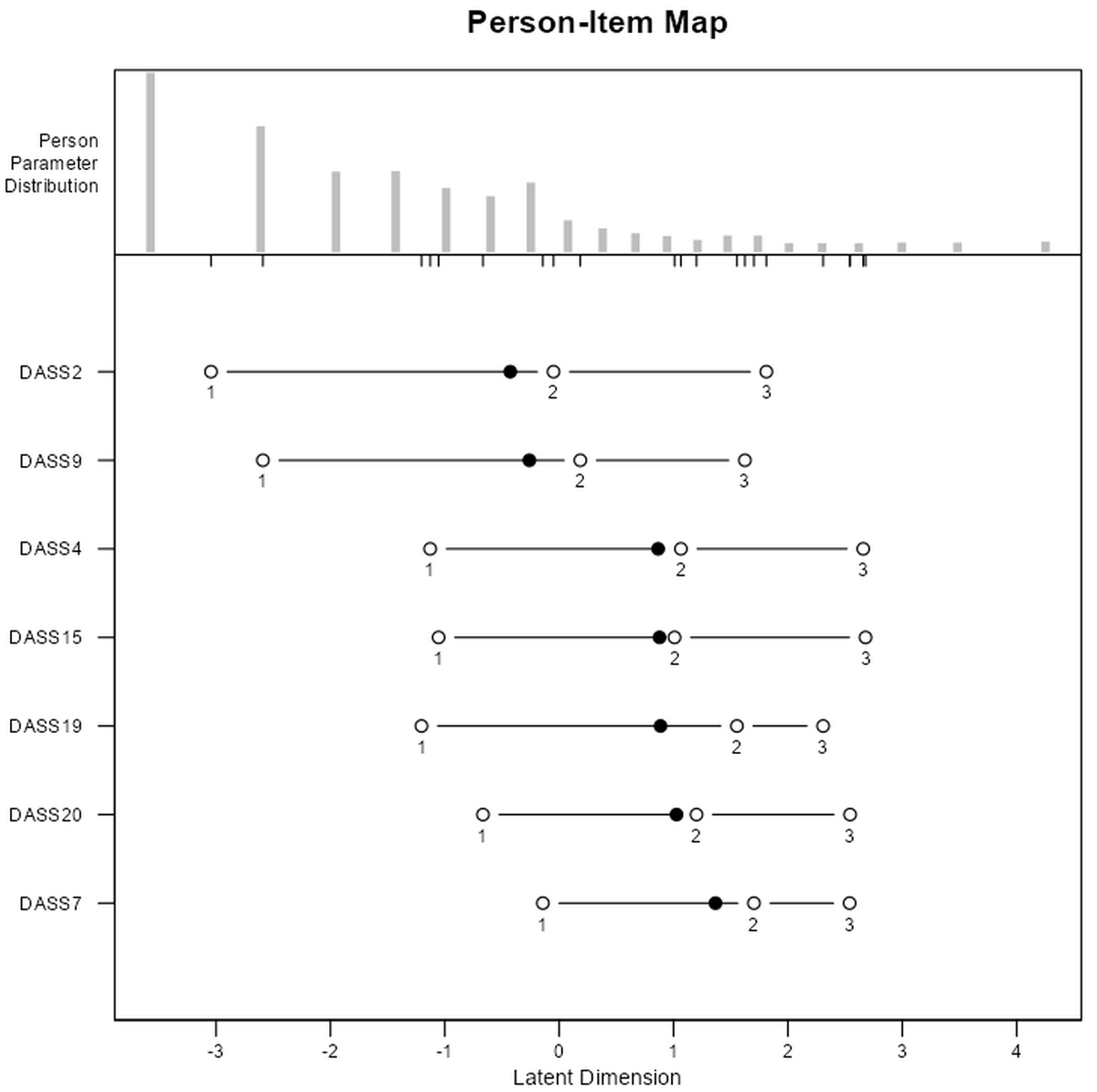 | 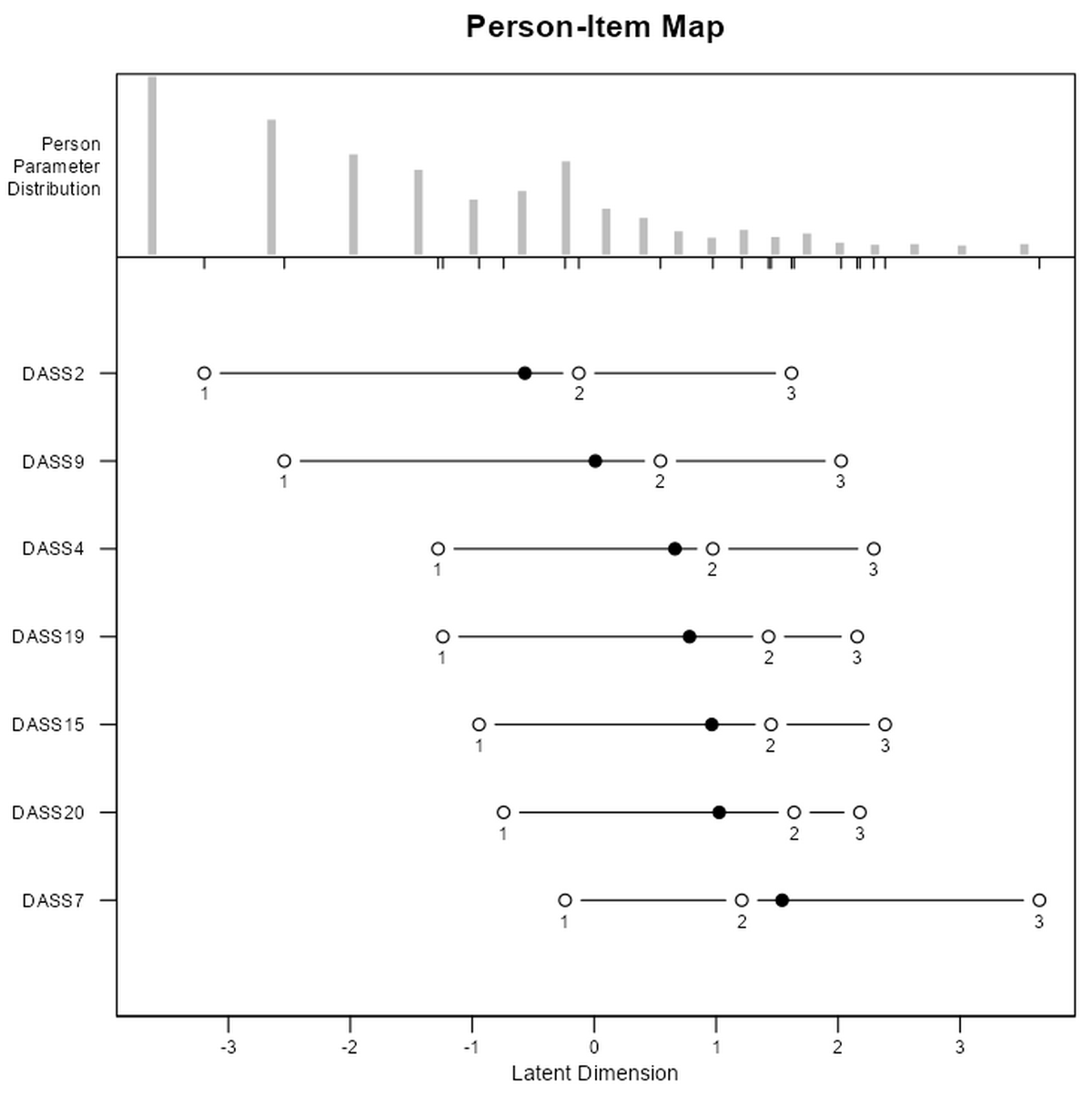 |
| --- | --- | --- |

**Figure S2.** Person-Item map of the anxiety subscale.

| 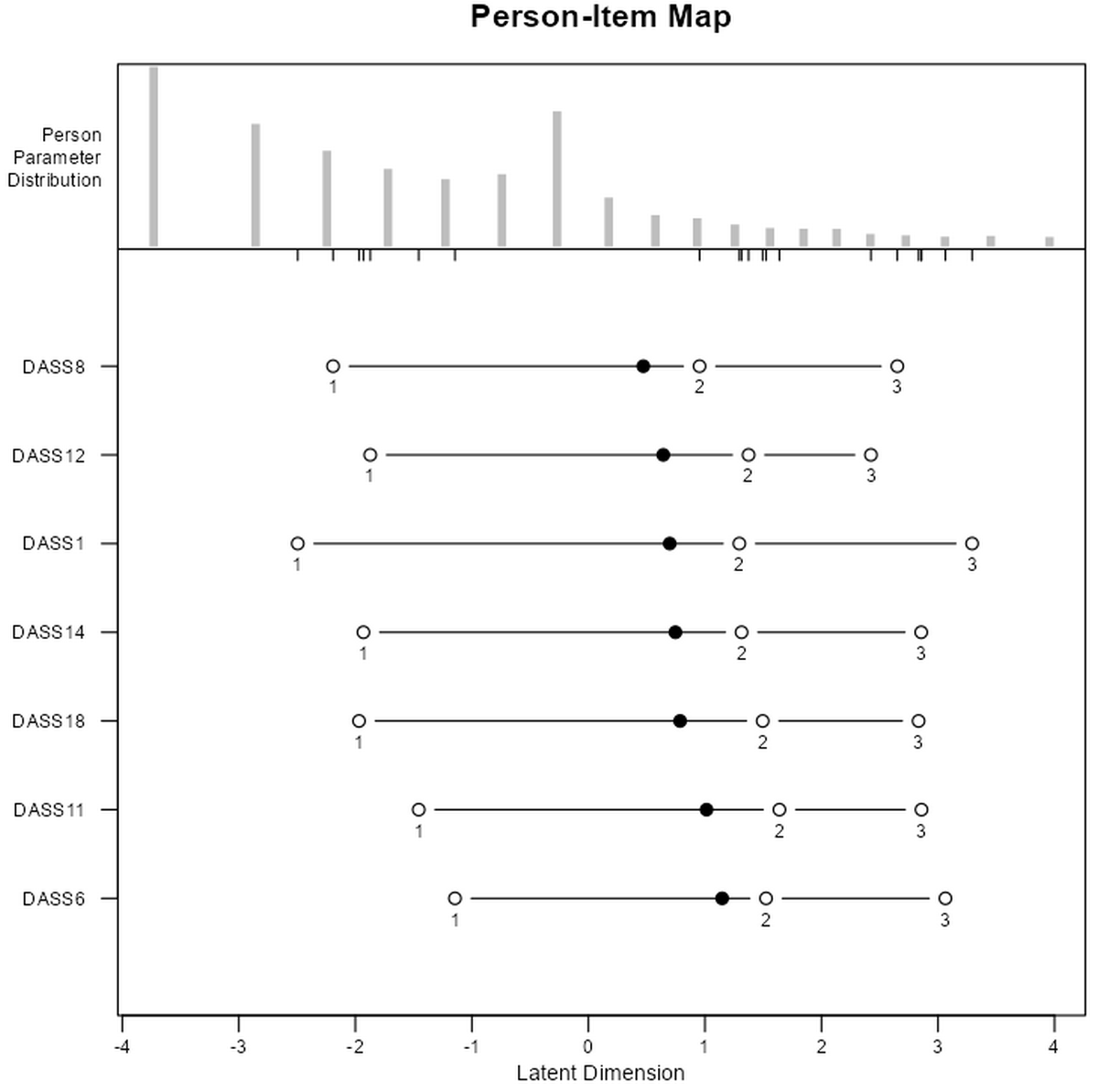 | 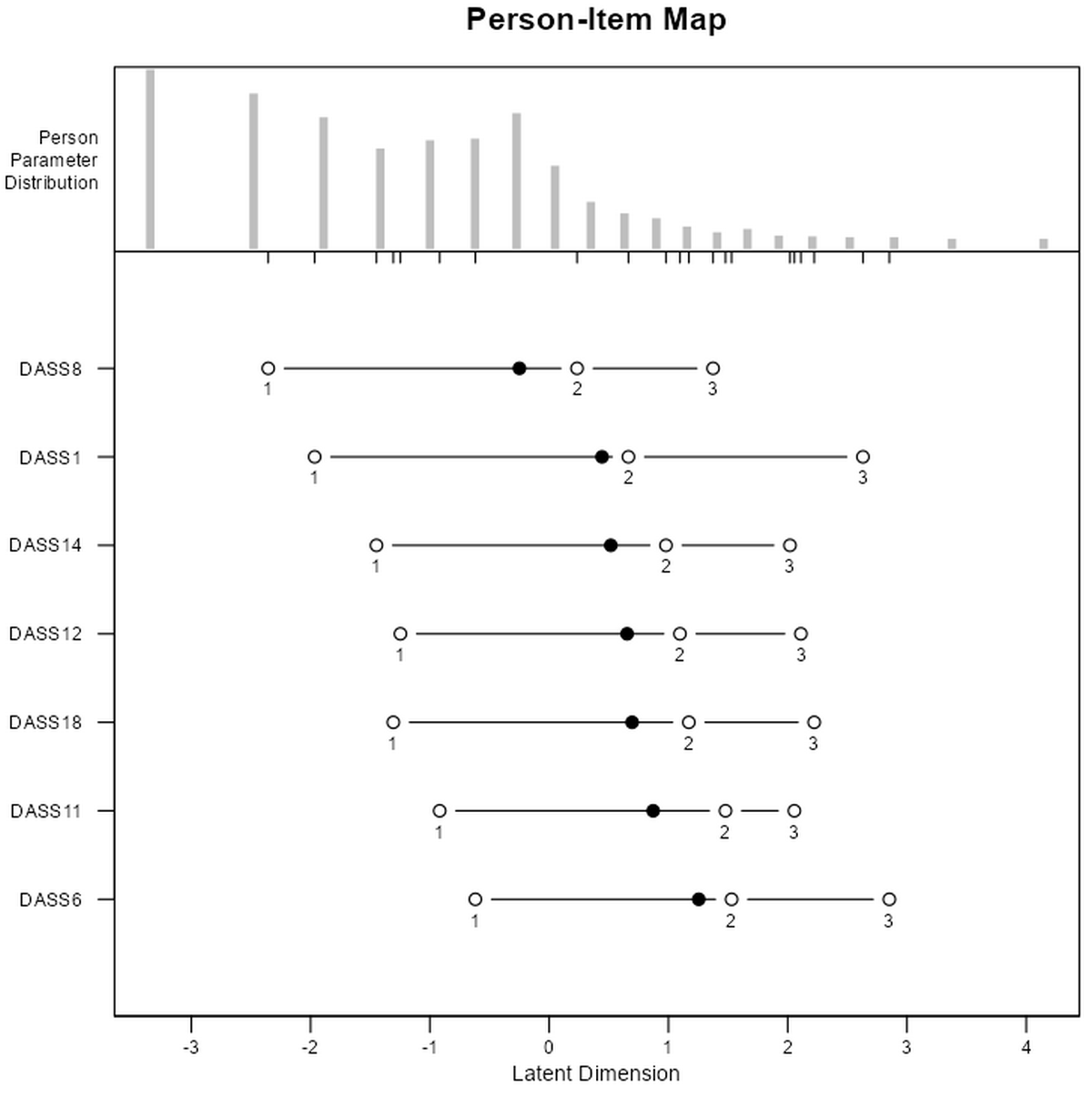 | 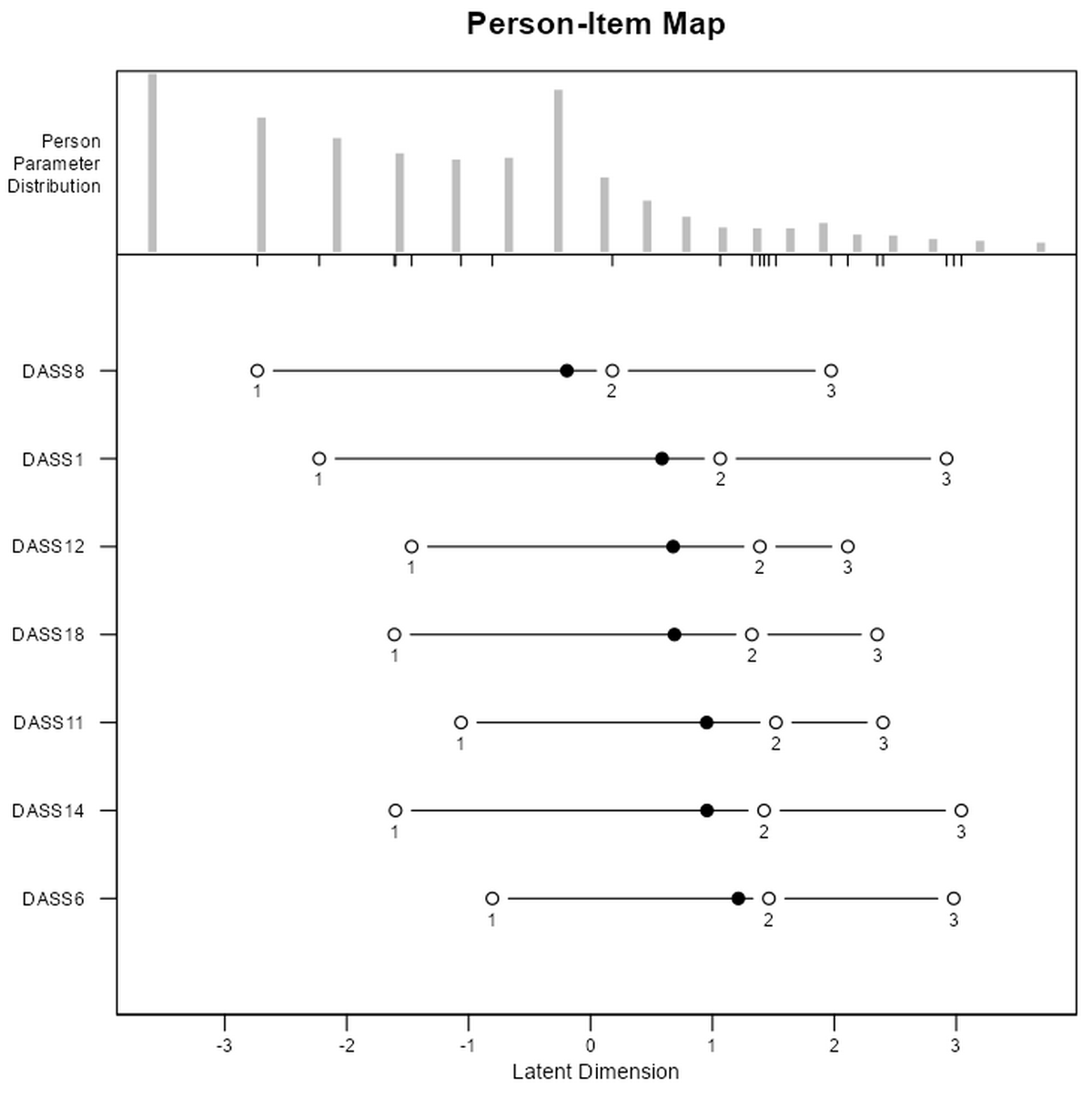 |
| --- | --- | --- |
| 2020 | 2021 | 2022 |

**Figure S3.** Person-Item map of the stress subscale.


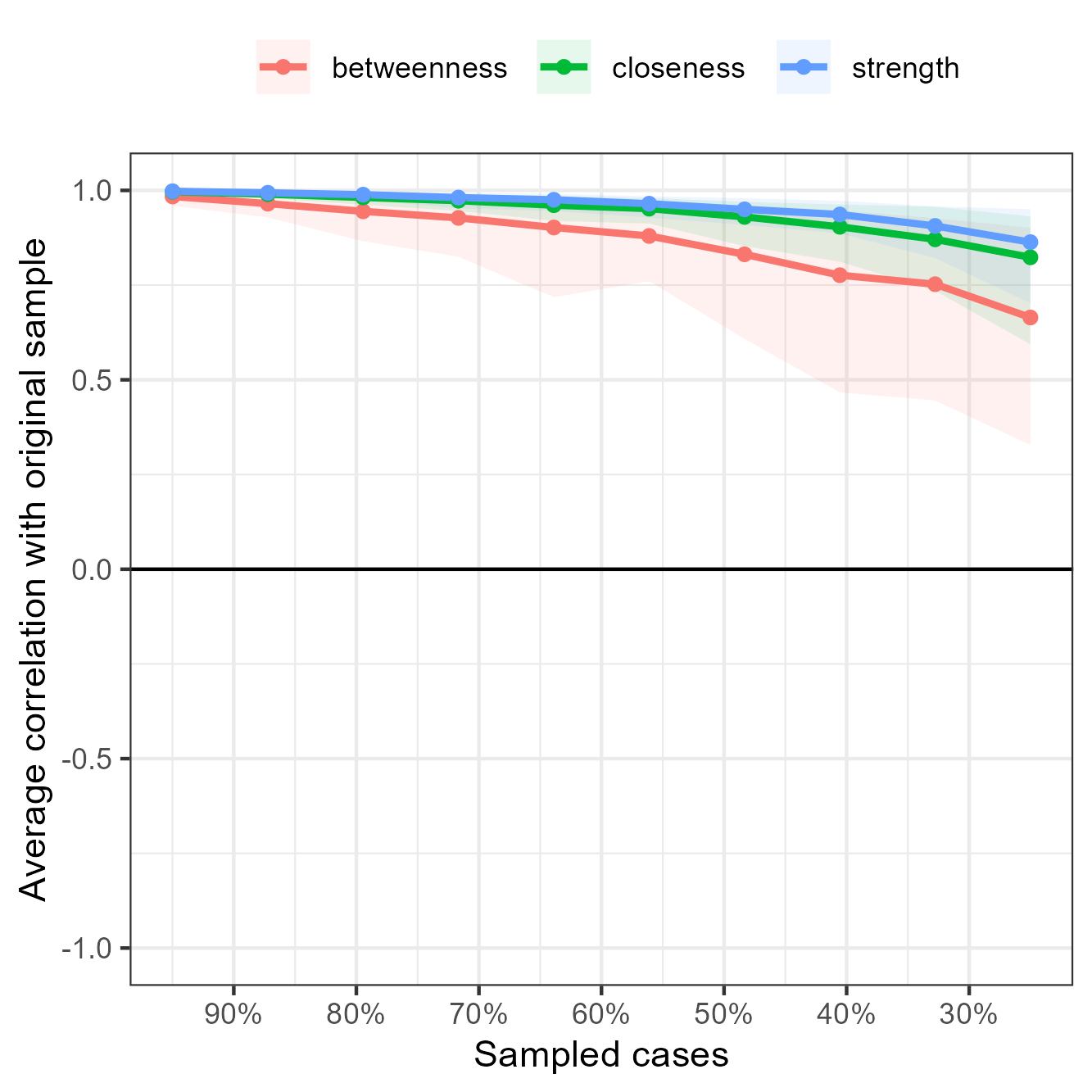


**Figure S4.** Network stability of the cross-sectional survey.


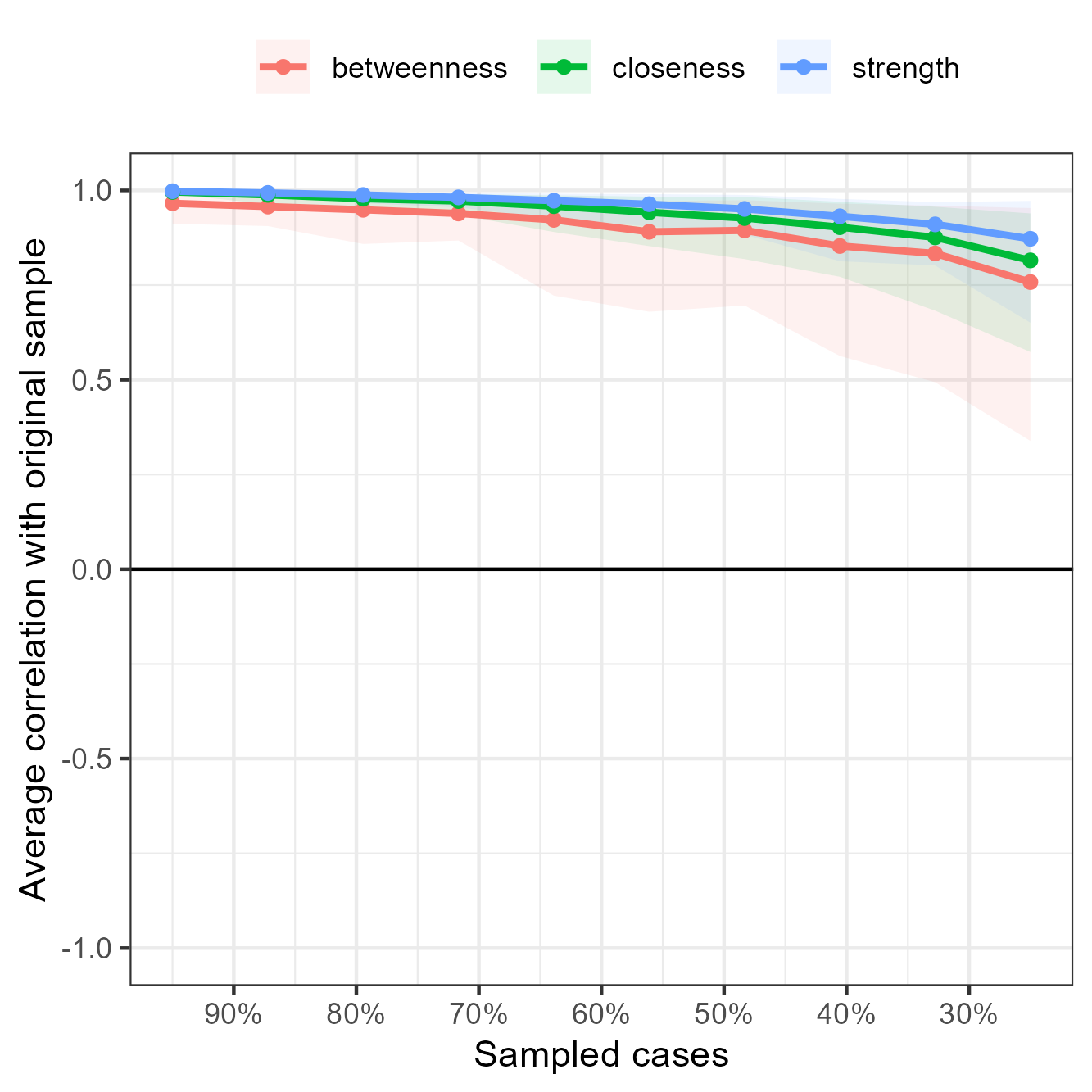


**Figure S5.** Network stability of the cross-sectional survey (revised).


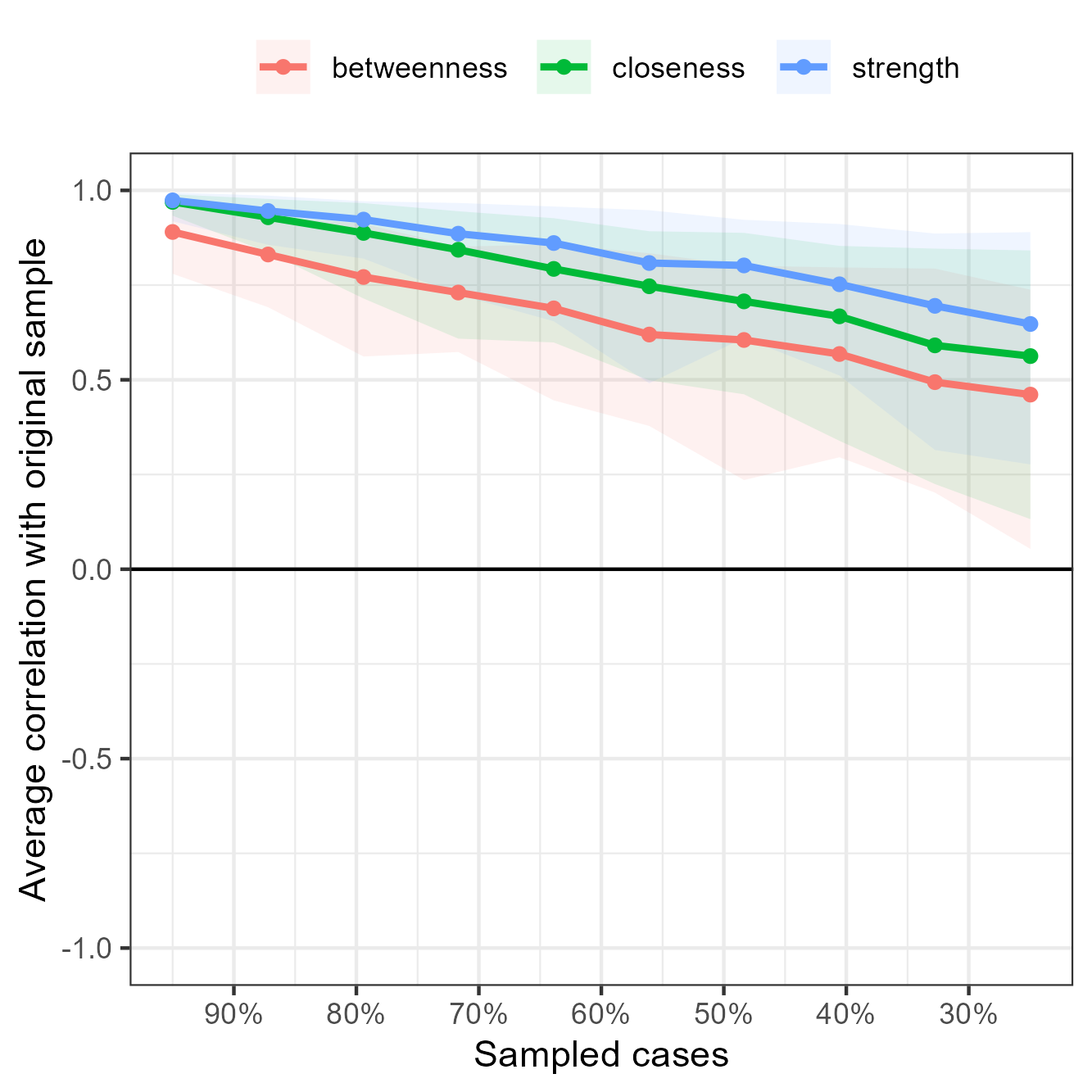


**Figure S6.** Network stability of the longitudinal survey (wave 1).


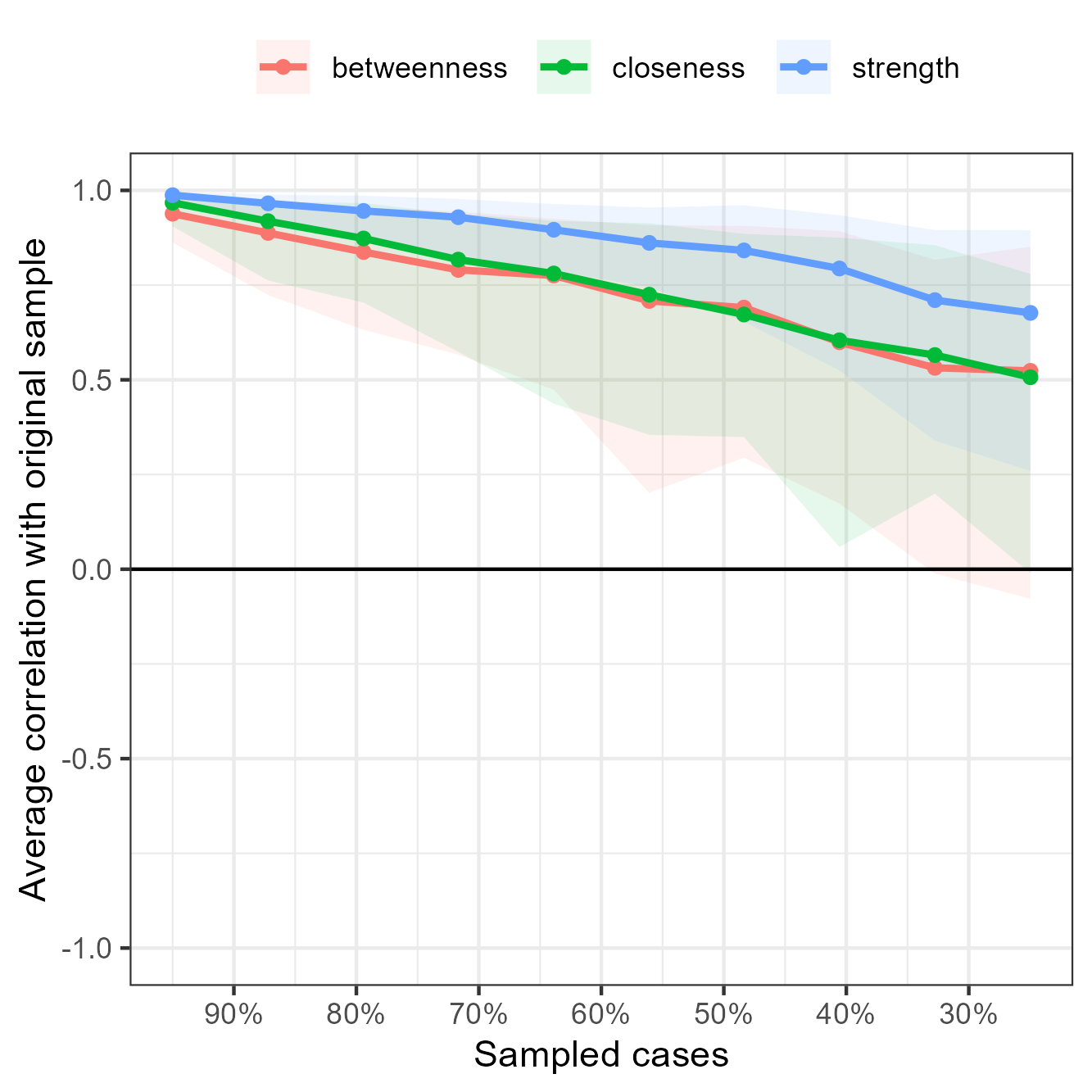


**Figure S7.** Network stability of the longitudinal survey (wave 1, revised).


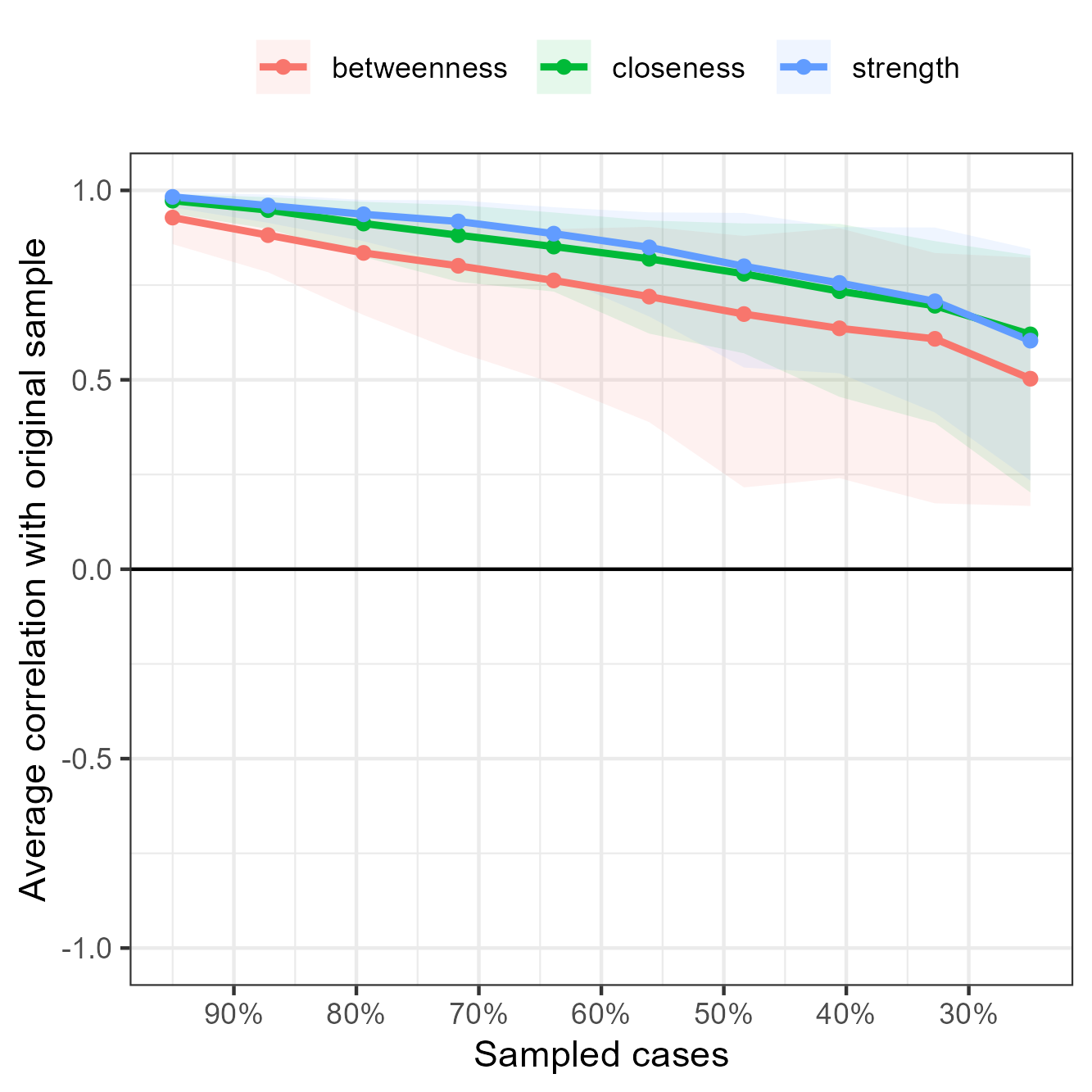


**Figure S8.** Network stability of the longitudinal survey (wave 2).


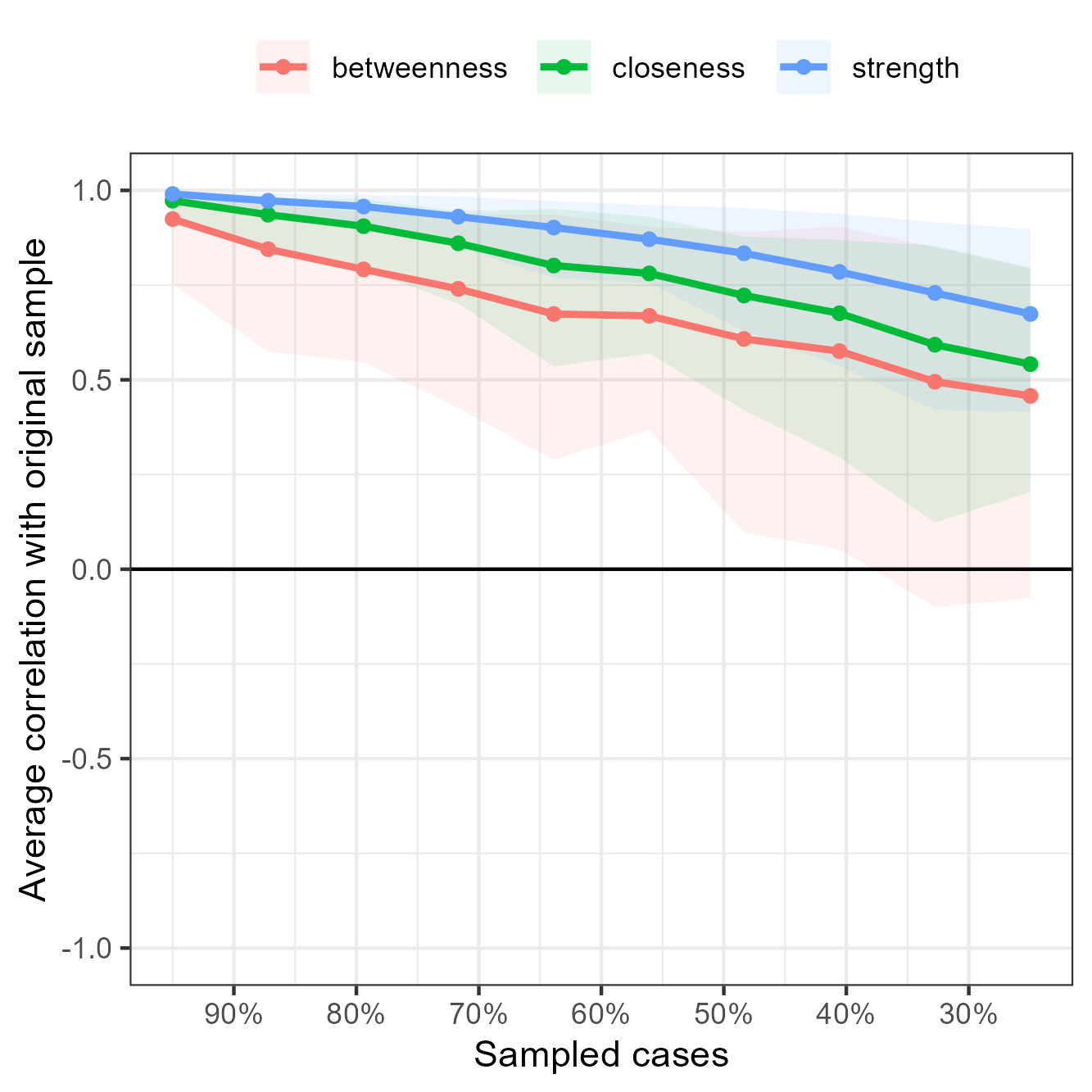


**Figure S9.** Network stability of the longitudinal survey (wave 2, revised).


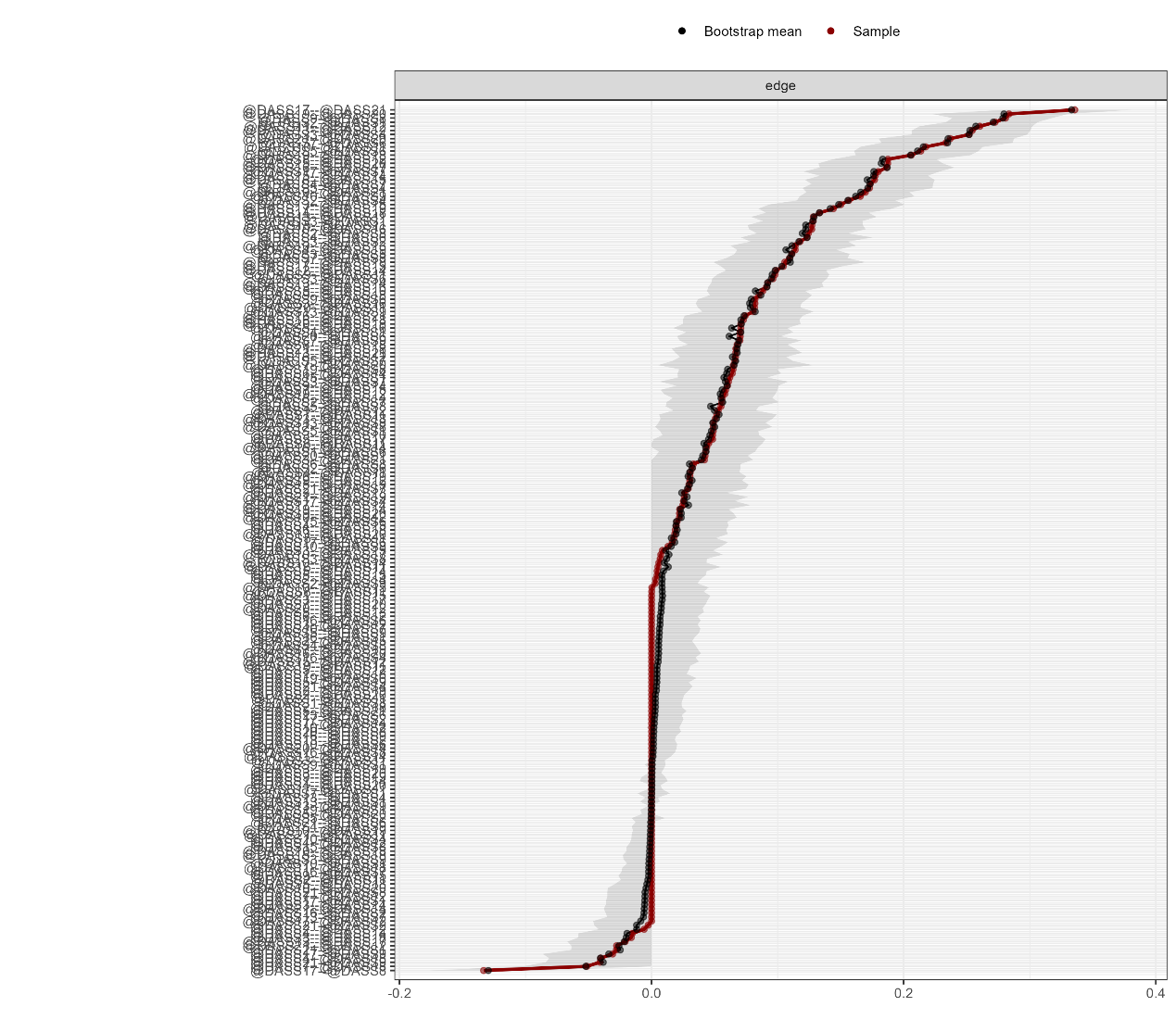


**Figure S10.** Network accuracy of the cross-sectional survey.


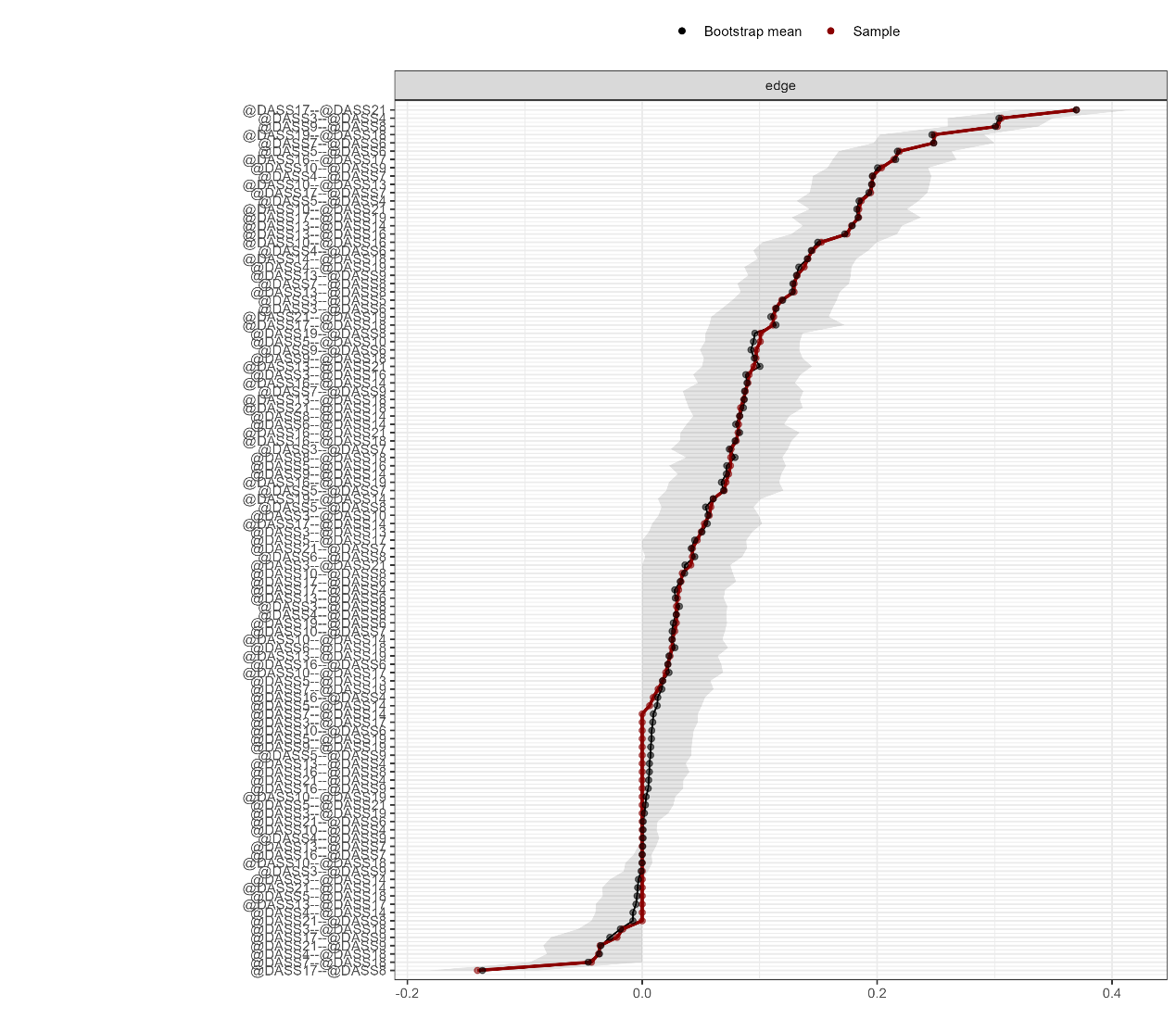


**Figure S11.** Network accuracy of the cross-sectional survey (revised).


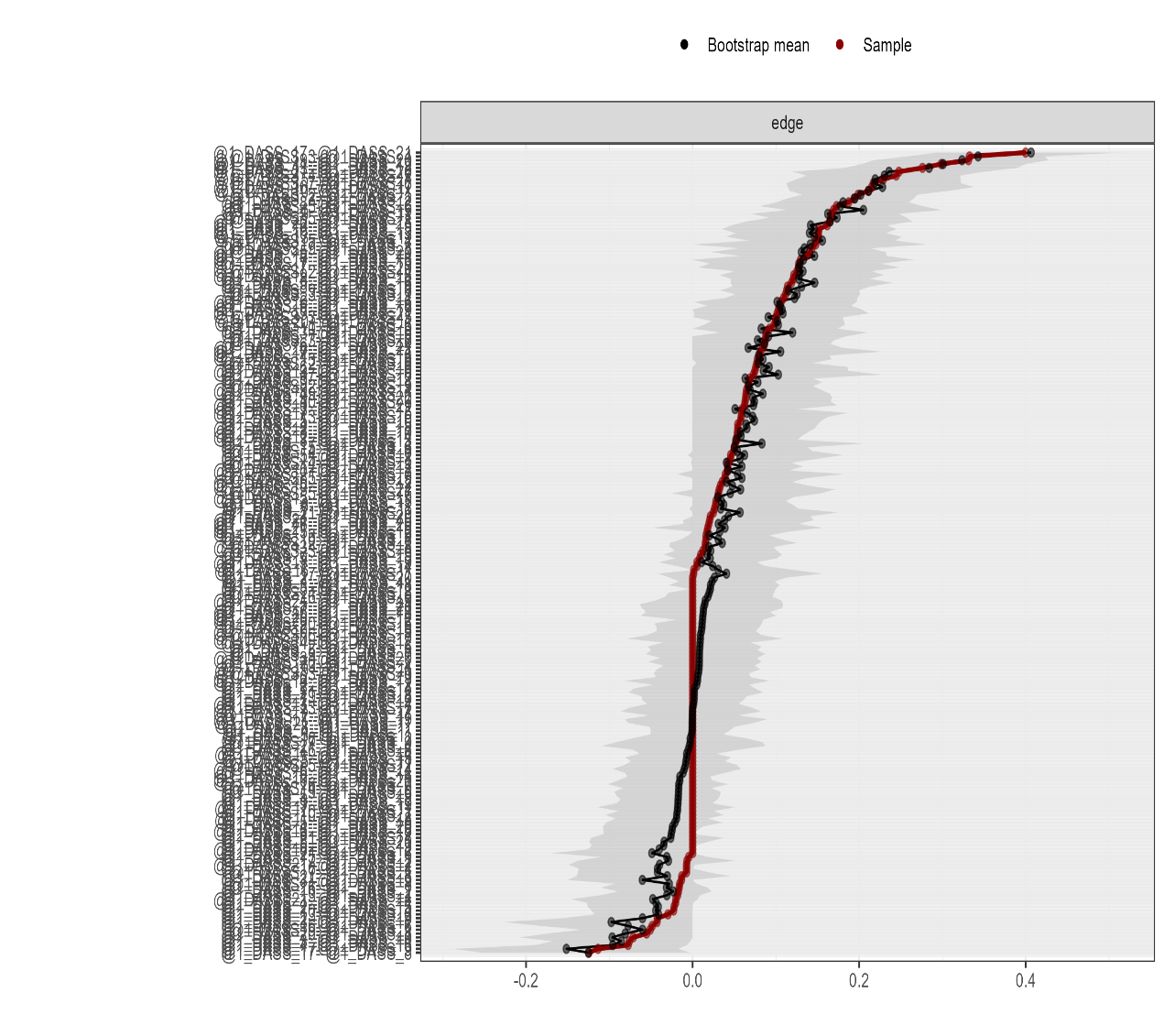


**Figure S12.** Network accuracy of the longitudinal survey (wave 1).


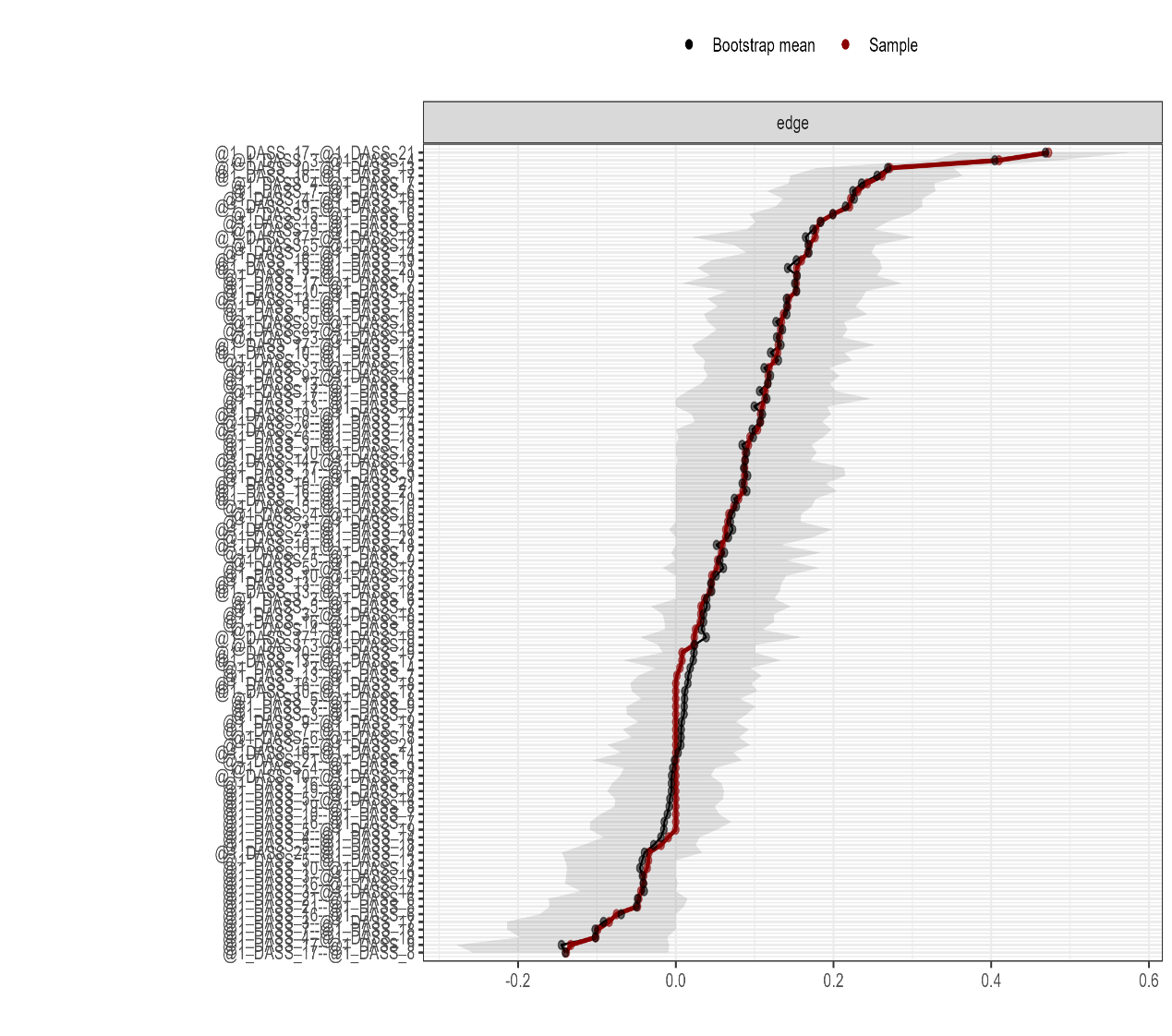


**Figure S13.** Network accuracy of the longitudinal survey (wave 1, revised).


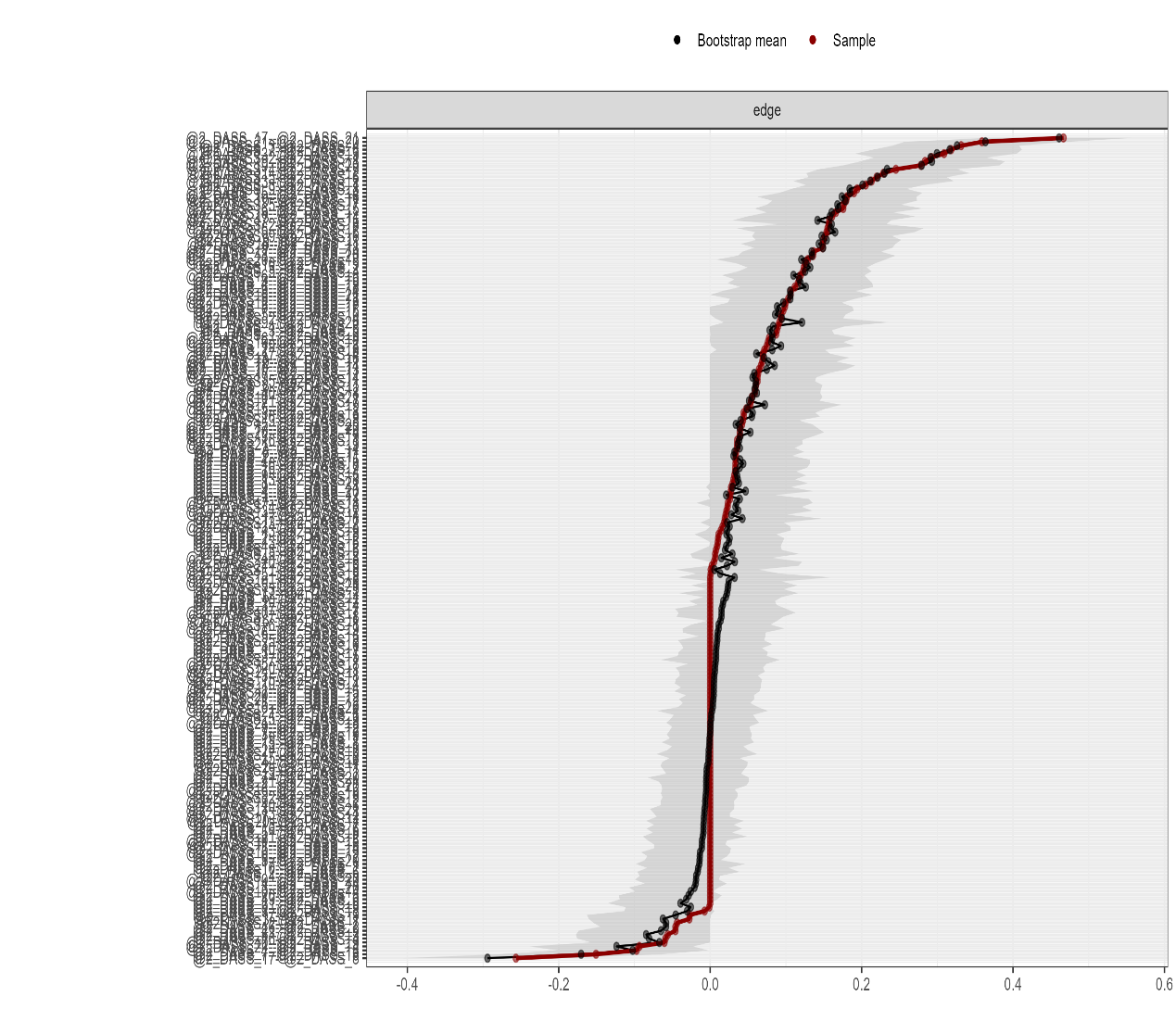


**Figure S14.** Network accuracy of the longitudinal survey (wave 2).


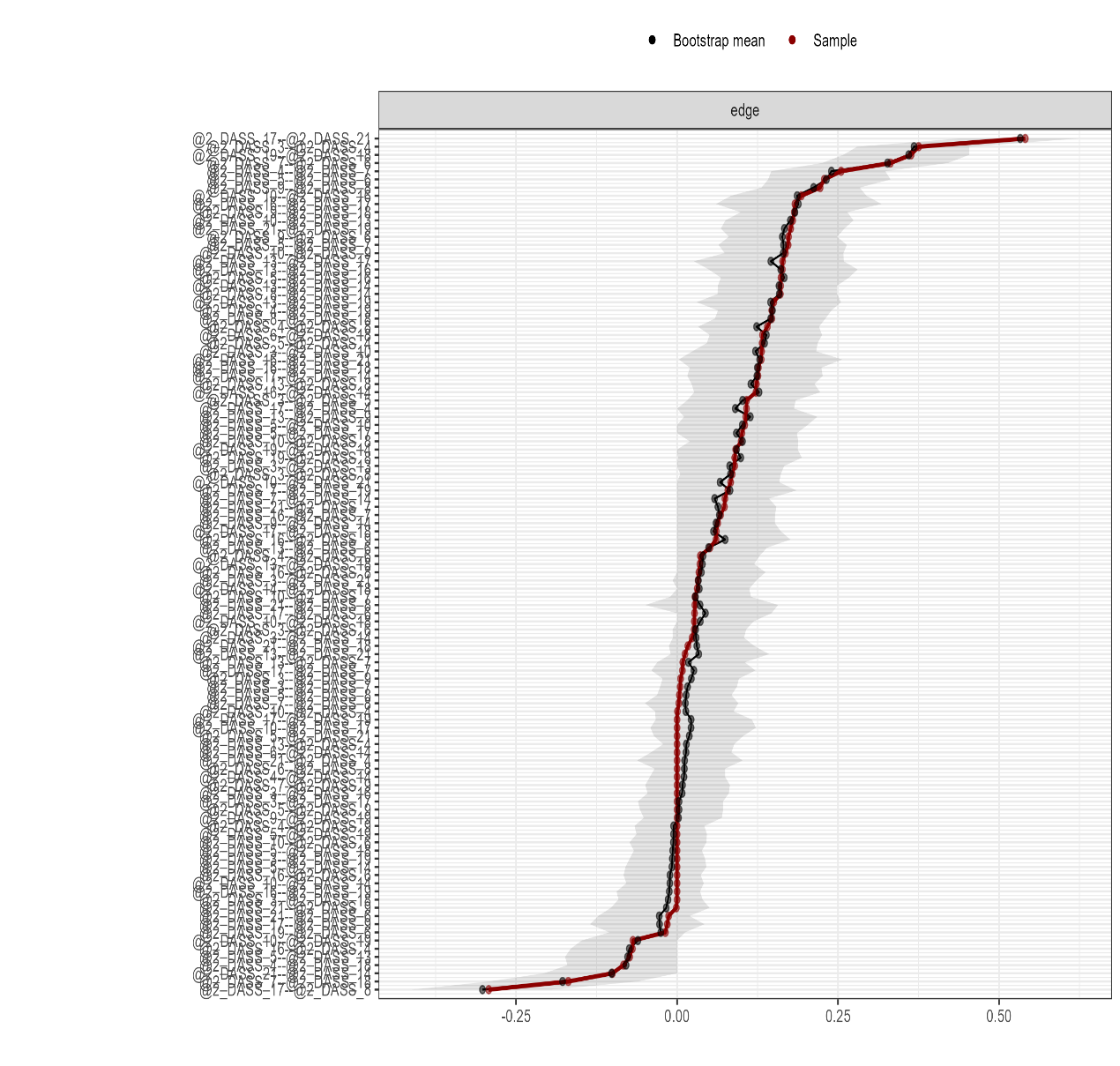


**Figure S15.** Network accuracy of the longitudinal survey (wave 2, revised).
